# Proteomic profiling reveals ACSS2 facilitating metabolic support in acute myeloid leukemia

**DOI:** 10.1038/s41417-024-00785-5

**Published:** 2024-06-08

**Authors:** Liliana H. Mochmann, Denise Treue, Michael Bockmayr, Patricia Silva, Christin Zasada, Guido Mastrobuoni, Safak Bayram, Martin Forbes, Philipp Jurmeister, Sven Liebig, Olga Blau, Konstanze Schleich, Bianca Splettstoesser, Thierry M. Nordmann, Eva K. von der Heide, Konstandina Isaakidis, Veronika Schulze, Caroline Busch, Hafsa Siddiq, Cornelia Schlee, Svenja Hester, Lars Fransecky, Martin Neumann, Stefan Kempa, Frederick Klauschen, Claudia D. Baldus

**Affiliations:** 1 Institute of Pathology, Ludwig-Maximilians-Universität München, Munich, Germany; 2 Department of Hematology and Oncology, Charité - Universitätsmedizin Berlin, a Corporate Member of Freie Universität Berlin, Humboldt-Universität, and Berlin Institute of Health, Campus Benjamin Franklin, Berlin, Germany; 3 Institute of Pathology Berlin, Charité - Universitätsmedizin Berlin, a corporate member of Freie Universität Berlin, Humboldt-Universität, and Berlin Institute of Health, Berlin, Germany; 4 Department of Pediatric Hematology and Oncology, University Medical Center Hamburg-Eppendorf, Hamburg, Germany; 5 Berlin Institute for Medical Systems Biology (BIMSB) at Max Delbruck Center for Molecular Medicine, Berlin, Germany; 6 German Cancer Consortium (DKTK), Partner Site Munich, German Cancer Research Center (DKFZ), Heidelberg, Germany; 7 Department of Proteomics and Signal Transduction, Max Planck Institute of Biochemistry, Martinsried, Bavaria Germany; 8 Department of Biochemistry, Oxford University, Oxford, UK; 9 Department of Hematology and Oncology, UKSH, Campus Kiel, Kiel, Germany

**Keywords:** Cancer, Cancer metabolism

## Abstract

Acute myeloid leukemia (AML) is a heterogeneous disease characterized by genomic aberrations in oncogenes, cytogenetic abnormalities, and an aberrant epigenetic landscape. Nearly 50% of AML cases will relapse with current treatment. A major source of therapy resistance is the interaction of mesenchymal stroma with leukemic cells resulting in therapeutic protection. We aimed to determine pro-survival/anti-apoptotic protein networks involved in the stroma protection of leukemic cells. Proteomic profiling of cultured primary AML (*n* = 14) with Hs5 stroma cell line uncovered an up-regulation of energy-favorable metabolic proteins. Next, we modulated stroma-induced drug resistance with an epigenetic drug library, resulting in reduced apoptosis with histone deacetylase inhibitor (HDACi) treatment versus other epigenetic modifying compounds. Quantitative phosphoproteomic probing of this effect further revealed a metabolic-enriched phosphoproteome including significant up-regulation of acetyl-coenzyme A synthetase (ACSS2, S30) in leukemia-stroma HDACi treated cocultures compared with untreated monocultures. Validating these findings, we show ACSS2 substrate, acetate, promotes leukemic proliferation, ACSS2 knockout in leukemia cells inhibits leukemic proliferation and ACSS2 knockout in the stroma impairs leukemic metabolic fitness. Finally, we identify *ACSS1/ACSS2*-high expression AML subtype correlating with poor overall survival. Collectively, this study uncovers the leukemia-stroma phosphoproteome emphasizing a role for ACSS2 in mediating AML growth and drug resistance.

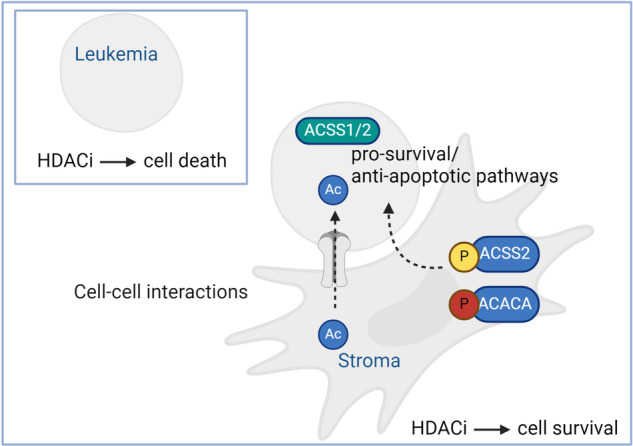

## Introduction

Individuals diagnosed with acute myeloid leukemia (AML) have a high risk for relapse of 20–80% [1]; however, factors that raise the risk of recurrence, apart from cytogenetic and molecular alterations, have been difficult to define. At the cellular level, drug resistance may be dependent on variations in genetic and epigenetic heterogeneity, drug metabolism, drug influx and efflux, oncogene reliance, and/or harboring inactivating mutations in apoptotic pathways [2–4]. Numerous studies have shown that in the microenvironment, both normal and AML-derived bone marrow mesenchymal stroma cells (BM-MSC) interact with leukemic cells and provide not only an optimal setting for AML persistence but also protection from therapeutic agents [4–10]. Thus, AML-stroma interactions play a critical role in AML progression, relapse, and persistence of leukemic blasts. Due to the highly proliferative nature of AML growth, a hypoxic microenvironment also helps reshape the way nutrients are metabolized to support increased biomass [11, 12]. The BM-MSC plays a pivotal role in providing metabolites, extracellular matrix, ligand/receptor binding, and signaling growth factors. These interactions are also imprinted on the aberrant gene and protein expression including cytogenetic background of BM-MSC from AML patients compared to healthy BM-MSC, reflecting the bidirectional effects in AML pathology [13–17]. Existing strategies aiming to interfere with pro-survival and anti-apoptotic signals from CXCR4/CXCL12, VLA4/VCAM-1, or fibronectin and CD44/hyaluronic acid ligand/receptor signaling are in development but far from clinical use [4, 18]. Thus, further research for determining how leukemic cells interact with stroma cells for leukemic growth or survival is critical in understanding relapsed states in AML.

Current proteomic research, including the analysis of post-translational modifications (PTMs), has been essential in identifying biomarker proteins key to leukemic cell survival, monitoring drug response, and disease stratification in AML [19–22]. Herein, we used an MS-based proteomic screening approach of bone marrow-derived primary AML (*n* = 14) to uncover a network of signaling proteins stimulated by AML-stroma interactions. Our findings show that among the significantly regulated proteins, a metabolic signature is one of the main enriched biological responses stimulated by AML-stroma interaction.

Recurrent genomic alterations in epigenetic modifiers are frequent in AML [23–26] thus epigenetic modifier drugs have been developed in combating resistant leukemic cells however with moderate success due to the difficulty in overcoming pro-survival/anti-apoptotic signaling. We surveyed AML drug sensitivities through an epigenetic drug screen targeting a wide array of epigenetic modifiers. We uncovered that leukemia protection was most pronounced in leukemia-stroma coculture following HDACi treatment, supporting the view that stroma-dependent epigenetic processes, may mediate drug resistance but also concomitantly link metabolic adaptations. Indeed, a quantitative phosphoproteomic approach was used to follow the stroma-dependent HDACi drug resistance response revealing that regulatory phosphosites in particular those associated with glycolysis and AMPK signaling pathways were significantly regulated. Among these players, an acetylation processing enzyme, ACSS2, and its substrate, acetate, stood out for the contribution to a leukemic proliferative response, increased metabolic fitness, and leukemic drug-resistant phenotype. Taken together, our findings provide valuable insights into ACSS2 functional role in AML-stroma interactions and suggest ACSS2 inhibition as a means to intervene in both epigenetic and metabolic support from the stroma.

## Materials and methods

### Cell culture and viability

HS-5 stroma and acute myeloid leukemia cell lines (KG1a, OCI-AML3, and K562) obtained from German Collection of Cell Culture, DSMZ, were cultured at 37 °C in a 5% CO_2_ incubator with standard cell culture reagents. For mono and coculture experiments, HS-5 stroma and leukemia cells were seeded separately at a density of 0.2 × 10^6^ cells/mL in RPMI plus 20% FCS and combined for coculture on the following day. Acetate (Sigma-Aldrich), Cytarabine (Ara-C, Cell Pharm, 20 g/mL), kinase inhibitors (Novartis), and Epigenetics Screening Library (Cayman chemical) were compounds used for treating leukemia cultures.

Primary AML bone marrow aspirates were obtained from patients enrolled in the Study Alliance Leukemia registry (refer to Supplementary Table 1 for cohort description). Bone marrow aspirates of AML patients and healthy donors were collected with informed consent in accordance with the declaration of Helsinki and approved by the Charité ethics committee. Mononuclear cells (MNC) were isolated using Ficoll-Paque density centrifugation. Fresh MNC fractions obtained from AML patients were seeded at 1–2 × 10^7^ cells/mL in StemPro-34 complete medium (Thermo-Scientific). In parallel, AML coculture blasts were seeded onto an 80% confluent stroma layer. Following 24 h, indicated drugs were added to cultures for apoptosis (48 h) and viability (48 h) measurements for primary AML and leukemia cell cultures in a 96-well format. A standard flow cytometer was used to measure positive cell populations of Annexin V-FITC (1:2000, BD Biosciences), a marker of early apoptosis. Stroma marker CD73-APC (1:2000, BD Biosciences) was used to discriminate stroma cells from leukemia cells in coculture experiments. Cell proliferation reagent WST-1 (Roche) was used to determine the viability of monocultures in untreated and treated conditions and measured with a TECAN spectrophotometer. Paired t-Test was used to generate p-values in order to compute the difference between monoculture and coculture apoptosis levels. P-values ≤ 0.05 were considered statistically significant. Standard western blotting using phospho-acetyl-CoA Carboxylase (Ser79, 1:1000, Cell Signaling) antibody was used for proteomic validation with β-actin (1:1000, Cell Signaling) as loading control. The protein lysates were resolved with 4–12% gradient gels (Thermo Scientific). For flow cytometry analysis of KG1a, immunostaining with histone antibody H3 (1 h, pacific blue conjugate, 1:100, Cell Signaling) and rabbit acetyl-histone H3 (1 h, Lys9, 1:250, Cell Signaling, referred to as H3K9ac) sequentially stained with goat anti-rabbit Alexa Fluor 488 plus (30 min, 1:500, Invitrogen) were used. KG1a were seeded at densities described before in a 96-well format and then treated with Api (HDACi, 1 µM, 48 h) or with acetate (10 µM, 48 h). Untreated leukemic cells, including unstained cells were used as base line for determining relative expression of untreated and treated samples. Paired T-test was used to calculate the significance difference between untreated versus treated treatments for each antibody.

### Primary AML and cell line sample preparation for shotgun proteomics

Freshly isolated primary bone marrow-derived AML (n = 14) were cultivated overnight at seeding densities between 1 × 10^7^ and 1 × 10^8^ cells as monoculture and coculture at 37 °C in a 5% CO_2_ incubator. Adherent HS-5 stromal beds were prepared 1-day prior at a density of 0.2 × 10^6^ cells/ml (at a 10–100 fold less density of AML cells). Suspended AML cells (Mono and Cocu AML, *n* = 28) were gently removed and re-seeded briefly onto a sterile plastic flask and placed into incubator for 30 min to allow contaminating adherent cells to re-attach. The suspended AML cells were then removed, centrifuged, washed with ice-cold PBS, and centrifuged at 300xg for 5 min at 4 °C. A 100 μl aliquot of the cell suspension was taken for FACS analysis to ensure that no detectable contaminating stroma (CD73-APC marker) was present. Lysis buffer was prepared with 7 M Urea, 2 M Thiourea, and 50 mM Tris-HCl at pH 8 containing Complete EDTA-free protease and PhosSTOP inhibitor cocktails (Roche Diagnostics GmbH). AML pellets were lysed in 0.2 mL volume and further processed for shotgun proteomic measurements. Leukemia cell line KG1a, stroma HS-5, and as a single unit, cocultured KG1a/HS-5 both treated (Api, 1 μM) and untreated cells were harvested and lysed as described above and were conducted in duplicate. An Api concentration and time point were pre-tested for an optimal non-apoptotic phase.

Samples were lysed on a Bioruptor sonicator (Diagenode), using 5 cycles of sonication (45 s ON, 15 s OFF). Protein concentration was determined by Bradford assay and 50 μg of protein of untreated KG1a, HS-5, coculture was reduced with 5 mM DTT for 45 min at 30 °C and alkylated with 22 mM iodoacetamide for 25 min at 25 °C. Proteins were digested using Lys/C (Wako, 1:40, w/w, overnight under gentle shaking, 30 °C) and immobilized trypsin beads (Applied Biosystem, 1:80, w/w/ 4 h under rotation, 30 °C). Before trypsin digestion samples were diluted four times with 50 mM ammonium bicarbonate. Digestion was stopped through acidification with 5 μL trifluoroacetic acid (TFA). Fifteen μg of each resulting peptide mixture was then desalted on Stage Tip [27], and the eluates dried and reconstituted to 15 μL in 0.5% acetic acid. HEK293 cell lysate was prepared with the same procedure and used for quality control.

LC-MS analysis for shotgun proteomics is described in detail in Supplementary Materials and Methods. Complete AML dataset containing label-free quantities determined by Max Quant software for each protein has been deposited to the ProteomeXchange Consortium via the PRIDE partner repository with the dataset identifier PD032051.

### Cell lysis and protein digestion for phosphoproteomics

HS-5 cells and KG1a were seeded at (0.2 × 10^6^ cells/mL) as described above. Api was used to stimulate stroma-induced protection at 1 μM for 5 h. Non-adherent KG1a monoculture cells (total of 5 × 10^7^) and cocultured cells (including adherent and suspended cells, a total of 2.5 × 10^7^) were washed with ice-cold PBS at 300xg and resuspended in urea lysis buffer; 2 mg of total protein was used for subsequent analysis as described previously [28]. Briefly, lysates were centrifuged at 20,800 × *g* for 30 min at 4 °C. Protein concentrations were determined using Bradford assays (Bio-rad). Proteins were reduced and alkylated with 10 mM DTT (dithiothreitol) and 30 mM IAA (iodoacetamide), followed by overnight trypsin (Promega) digestion at a ratio of 1:20. The digest was acidified using formic acid and particulates were removed by centrifugation for 30 min at 2500 × *g*.

### Stable isotope dimethyl labeling

Peptides were desalted and dimethyl labeled on a column as previously described with slight modifications [28]. OASIS HLB (Waters) cartridges were washed and conditioned with acetonitrile (ACN) and 0.1% formic acid. After sample loading peptides were washed with 0.1% formic acid and labeled with “light” (CH_2_O (Sigma) + NaBH_3_CN (Fluka)), “medium” (CD_2_O (Isotec) + NaBH_3_CN) or “heavy” (13CD_2_O (Isotec) + NaBD_3_CN (Isotec)) labeling reagent. Following washing, labeled peptides were eluted with 0.1% formic acid/80% ACN and pooled according to Table 1.

**Table 1:** Experimental parameters and isotope labeling scheme used in determining phosphorylation networks of leukemia (KG1a), stroma (HS-5), and KG1a/HS-5 coculture.

Experiment	Experiment ID	Lysate Content			Total Phosphosites			Experiment-Reference		Sites Above Threshold^a^	
		Light	Medium	Heavy	Light	Medium	Heavy	Ratio 1	Ratio 2	Ratio 1	Ratio 2
Leukemia-stroma	Mix 1	HS5	KG1a	CoCu	741	593	727	CoCu : HS5	CoCu : KG1a	287	412
HDACi-induced stroma protection	Mix 2	KG1a*	CoCu*	CoCu	2389	2381	2388	CoCu* : KG1a*	CoCu* : CoCu	684	683
HDACi-induced stroma protection	Mix 3	HS5*	CoCu	CoCu*	695	682	676	CoCu* : HS5*	CoCu* : CoCu	156	169

^a^Experimental parameters and isotope labeling scheme are described including the significant phosphosphosite residues detected in leukemia (KG1a), stroma (Hs5), and KG1a-Hs5 coculture (Cocu). Stroma induced protection treated wtih HDACi, Api, for 5 h at 1 mM are annotated by a single astrix. The number of total phosphosites detected for each lysate mix (Mix 1, 2 or 3) are listed under columns Light, Medium, Heavy. The intensity values at each phosphosite above threshold was determined by determining the fold change (Experiment-Reference) and termed ratio 1 (heavy:light) and ratio 2 (heavy:medium). The significant number of phosphosites above threshold are values of ±1.0 (with exception of experiment Mix 2, a higher threshold was set at ± 2.0).

### Phosphopeptide enrichment

Enrichment of phosphorylated peptides was performed using a previously described TiO_2_ enrichment protocol [29]. Briefly, 40 µL of 500 mg/mL Titansphere TiO (GL Sciences Inc.) in 80% ACN/0.2% TFA) was washed with 0.6% ammonia solution (Merck) and sample buffer (80-mg/mL glycolic acid/80% ACN/2% TFA solution). 2x sample buffer was added 1:1 to labeled peptides solutions and centrifuged for 10 min at 16,000x g. Then peptides were sequentially incubated with 500 mg/mL TiO_2_ (2 h and 1 h) and 100 mg/mL TiO_2_ (overnight, 1 h, and 1 h) for 5 incubation steps. After loading TiO_2_-peptide solution onto a TiO_2_ micro-column, the beads were washed with sample buffers 80% ACN/0.2%TFA and 20% ACN. Consecutive elution of phosphopeptides was executed by three times addition of 30 µL of 0.6% ammonia solution and acidified with 0.9% TFA. All eluents from the same sample were pooled, concentrated in a SpeedVac, and stored at −80 °C. Samples were desalted using C18 tips (Thermo Scientific) according to the manufacturer’s instructions. Mass spectrometry and data preprocessing for phosphoproteomics are described in Supplementary Materials and Methods.

### Statistical analysis of AML datasets

AML RNA expression datasets from TCGA [30] was used to determine the impact of metabolic gene expression in AML. The TCGA RNA-seq dataset was used as processed in Pancan12 [24], and imported into Gitools 2.3.1 [31, 32] to examine differential gene expression. Group comparisons and correlations between specified groups of patients were calculated using the table By, arsenal, and ‘survival’ packages in R. Analyses are further described in Supplementary Materials and Methods.

### CRISPR Cas9 gene editing

Following Zhang et al. [33] methods for genomic editing, Cas9 expression plasmid was obtained from Addgene (pX458) and WTSI genome editing tool was used to design gRNA for human ACSS2. Two flanking sgRNA target sequences were selected for removing 178 bp of exon 1 containing S30 ACSS2. ACSS2-SgRNA1 5′-CACCgTCTAGGAACTTGACGTGATG was annealed to ACSS2-SgRNA2 5′-AAACCATCACGTCAAGTTCCTAGAc, and ACSS2-SgRNA2 5′-CACCGCGCTCCGTGGAGGAGCCGC was annealed to 5′-AAACGCGGCTCCTCCACGGAGCGC. Transfected p485/gRNA1 and p485/gRNA2 leukemia cells were FACS sorted in 96/well plates (single cell), and clones were expanded and characterized by quantitative PCR (Sybr Green, Fermentas), DNA sequencing, and western blotting using ACSS2 antibody (1:1000, Cell Signaling).

### Oxygen consumption rate

KG1a, OCI-AML3, and K562 were examined for mitochondrial respiration capacities with Mito Stress Assay (Seahorse Bioscience). Experiments were conducted according to the manufacturer’s instructions. Monoculture leukemic cells and pre-cocultured leukemia cells with HS-5 WT or with HS-5 ACSS2-KO cells were cocultured overnight as described above before the assay test. Leukemia cells from both mono- and pre-cocultured leukemia cells (0.2 × 10^6^ cells/mL) were removed and washed twice with 1 mL RPMI XF base medium Supplemented with 10 mM glucose, 2 mM glutamine, and 1 mM sodium pyruvate at pH 7.4. Cells were pre-equilibrated in media for 1 h at 37 °C and in a 5% CO_2_ incubator. Cells were re-seeded in RPMI XF base medium (optimal at 0.4 × 10^6^ cells/mL) into the provided manufacturer’s microwell plate and incubated for 45 min in a non-CO_2_ incubator at 37 °C to again pre-equilibrate to reduced oxygen levels. Oxygen consumption rate (OCR) was measured through the sequential addition of 0.2 µM oligomycin (Oligo), 0.25 µM Carbonyl cyanide-4-phenylhydrazone (FCCP), 5 µM rotenone, and 5 µM antimycin A (Rot/Anti). Following calibration, the XF96 analyzer 4-port injection standard protocol was followed. Seahorse XF Analyzer measured the OCR of live cells in a 96-well plate.

## Results

### AML-stroma proteome unveils a protein network mainly associated with metabolic function

**Fig. 1:**
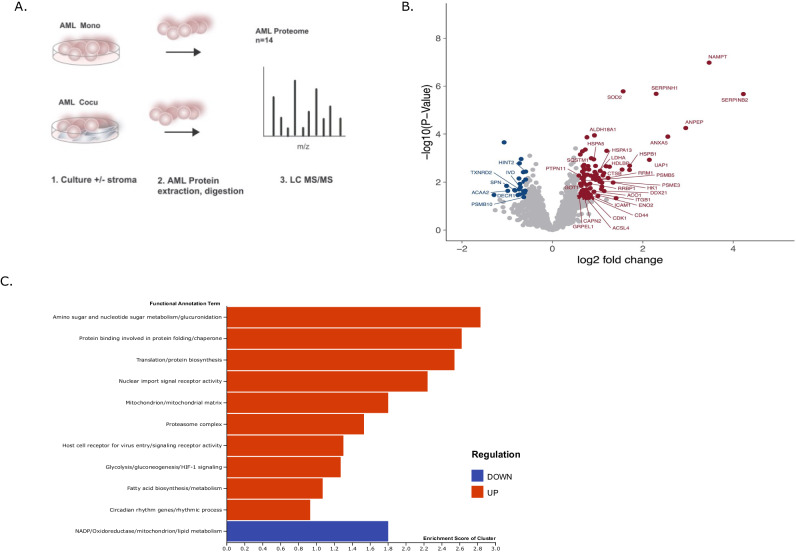
The proteomic profiles of AML samples are metabolically impacted by the interactions with BMSC. **A** A schematic diagram depicts how AML protein extracts were created from 14 primary AML cultured overnight in the presence and absence of HS-5 stroma cells, referred to as AML Mono and AML Cocu, respectively. Both AML Mono (*n* = 14) and AML Cocu (*n* = 14) were collected and protein lysates were processed for LC-MS/MS to detect proteins involved in signaling pathways stimulated by stroma. **B** A volcano plot depicts the differential protein expression in AML Cocu with a ≥1.5 fold change relative to AML Mono. Upregulated, downregulated, and proteins below the cutoff of 1.5 fold change are displayed in red, blue, and gray, respectively. **C** A bar plot depicts top biological pathways that are activated in primary leukemic cells in response to stroma interaction (Supplementary Dataset 1).

BMSCs support leukemogenesis and allow leukemic cells to evade chemotherapy-induced cell death; however, the protein-signaling network leading to leukemic growth and cell survival remains unknown. To unravel early (pre-apoptotic) changes in protein abundance regulated by leukemia-stroma interactions, we cultured primary AML (*n* = 14) in the absence (AML Mono) or presence of HS-5 stroma cells (AML Cocu) and measured an untargeted global proteomic profile using LC-MS/MS (Fig. 1A, Supplementary Table 1, and Supplementary Dataset 1). A summary of patient clinical information such as gender, age, cytogenetics, and mutation status is presented in Supplementary Table 1. Overall, the average protein counts in each sample AML Mono (2359 ± 430) and AML Cocu (2540 ± 333) were equally represented. A total of 2231 unique proteins were identified and quantified. We determined the differentially expressed proteins with a fold change of >1.5 (FDR 0.05 cutoff) between AML Cocu and AML Mono (Fig. 1B) of which 114 proteins were significantly differentially regulated. In comparison to HS-5 monoculture, a minimal overlap of proteins was unique to stroma. To gauge molecular responses triggered by AML-stroma interaction, gene set analysis shows an upregulation of membrane and cell differentiation markers including ICAM1, ITGB1, SPN, ANPEP, and CD44. The differentially regulated proteins were also subjected to DAVID and ClueGo for enriched molecular pathways. Thirty-eight of 114 proteins are associated with upregulation of metabolic processes and associated with enrichment of nucleic acid metabolism, translation/protein biosynthesis, mitochondrial matrix, glycolysis and gluconeogenesis, fatty acid biosynthesis, and signaling receptor activity (Fig. 1C). Rate limiting NAD biosynthesis factor NAMPT, related to energy metabolism, was the most stroma-dependent upregulated protein across the 14 AML samples. Down regulated proteins (25/144) were enriched for NADP, oxidoreductase, mitochondrion, and lipid metabolism. The primary AML cells stimulated by stroma interaction reveal a proteomic network predominately representing a signature of enriched metabolic pathways including an upregulation of early membrane signaling molecules.

### Stoma-dependent protection of leukemic cells was most evident with HDAC inhibitor treatment

Recurrent mutations of epigenetic modifiers that appear in pre-relapse and post-relapse AML cases indicate the importance of understanding epigenetic modalities in drug resistance [34, 35]. AML-stroma interactions also lead to increased drug resistance that might be in part dependent on deregulation of leukemic epigenetic pathways. Thus to further understand pro-survival/anti-apoptotic response in AML, we modulated epigenetic pathways to give insight into how leukemia becomes resistant through stroma interactions [36, 37]. To this end, we performed an epigenetic drug screen targeting a wide array of epigenetic modifiers (80-epigenetic compound library). First, proliferative growth was measured for each compound in 9 leukemia cell lines. The compound library was then clustered by drug target function, which included histone deacetylase inhibitors (HDACi), histone methyltransferases (HTMi), bromodomain (BETi), DNA methylase (DNMTi), and sirtuins (SIRTi). Inhibitors targeting histone deacetylases significantly reduced cell growth across all 9 leukemia cell lines relative to untreated cells, indicating that targeting histone acetylation may achieve the greatest effect on leukemic subtypes (Supplementary Fig. 1).

**Fig. 2:**
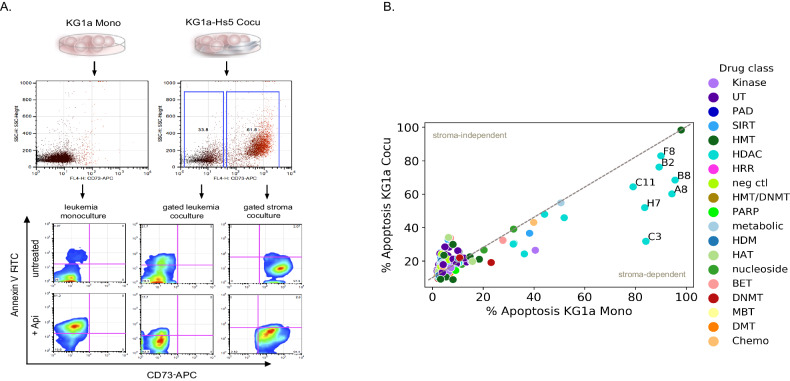
The drug sensitivity differences between AML Mono and AML Cocu identify that HDACi compounds promote stroma-dependent protection of leukemia. **A** A schematic diagram is depicting the flow cytometer gating approach for monitoring apoptosis levels in KG1a Mono and KG1a-HS-5 Cocu. CD71-APC was used to label stroma (HS-5 cells) thus CD71-APC negative cells trace leukemia cells. Annexin V-FITC was used to determine the extent of apoptosis. An example is given of stroma protection of leukemia cells treated for 48 h with HDACi, Api (10 µM). The apoptosis levels of KG1a Mono (4%) and KG1a-HS-5 Cocu (21%) untreated are compared with KG1a Mono HDACi treated at 81% versus KG1a-HS-5 Cocu at 17.7%, respectively. **B** Summary of apoptosis levels in KG1a Mono and KG1a Cocu following treatment with an 80 epigenetic compound drug library. The x and y-axes represent the percent of apoptosis levels in KG1a Cocu and KG1a Mono, respectively. The colors indicate drug classes and were at a 10 μM concentration for each compound. The plot depicts the significance of at least 3–6 replicate measurements of the data where the labeled points indicate ≤0.05 *P* Value determined by a paired t-test. The gray dash distinguishes the slope of 1 where less than 1 signifies % apoptosis coculture is less than monoculture (stroma-dependent protection trend). Supplementary Dataset 2 lists the compound description used in this study.

To model leukemia-stroma interactions, we then determined the effect of apoptosis on leukemic KG1a cells in monoculture (KG1a Mono) versus KG1a coculture with stroma HS-5 cells (KG1a-HS-5 Cocu). We treated KG1a Mono and KG1a-HS-5 Cocu again with the 80 epigenetic compound library for 48 h (Fig. 2A, B). Treatment with histone deacetylase inhibitors (10 μM), in particular Apicidin (C3), M344 (A8), CBHA (H7), SAHA (C11), and Oxamflatin (B8) significantly induced apoptosis in KG1a monocultures, whereas in coculture apoptosis was reduced by 10–50%. Of the 30 HDACi compounds surveyed, these five compounds primarily target HDAC Class I. Stroma-induced protection of leukemia cells was most evident with Apicidin (Api) treatment, which effectively induced apoptosis in monocultured leukemia (84%) than in cocultured leukemic cells (31%, Fig. 2A, B). The protective properties of stroma were further verified by coculture and treatment of five HDACi (A8, B8, C3, C11, and H7) in other leukemia cell lines and primary AML (*n* = 10, Supplementary Fig. 2). In 6 of 10 AML primary cocultures, at least 40% apoptosis levels were reached in monoculture with HDACi treatment but exhibited significantly reduced apoptosis levels in coculture. These results indicate that cocultured leukemic cells treated with HDACi may be protected from HDACi cytotoxicity in a stroma-dependent manner.

### Identification of phosphorylation events as stroma protective effects

**Fig. 3:**
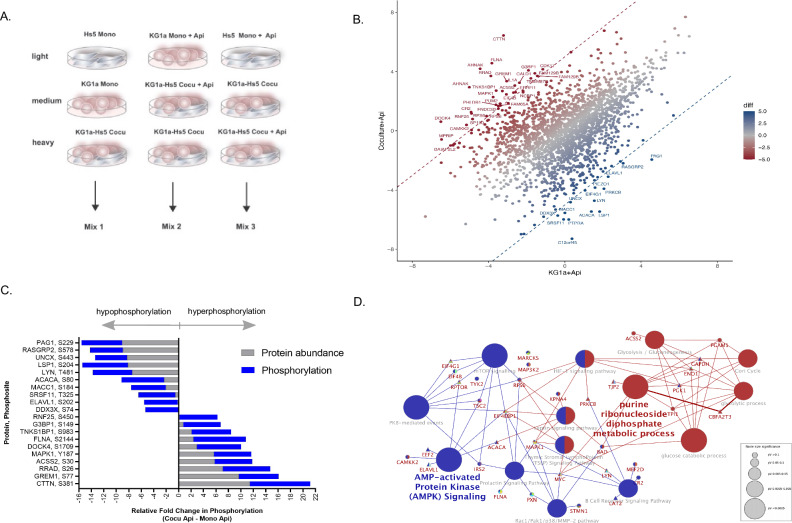
The leukemia-stroma phosphoproteome reveals biological pathways leading to stroma-dependent protection of leukemia cells. **A** Discovery phosphoproteomic tools were used to identify and determine relative amounts of phosphorylated phosphosites in KG1a Mono, HS-5 Mono, and KG1a-HS-5 Cocu. Briefly, following a 24h-culturing period, cells were treated with HDACi Api at 1 μM for 5 h, a sub-apoptotic time point, and then harvested to create protein lysates. Protein extraction of each lysate, digestion, and isotope labeling was performed on each experimental condition. To compare phosphosite peptide intensities between culturing conditions, a multiplex labeling scheme of light, medium, and heavy-labeled lysates were used. Light, medium, heavy labeled lysates were pooled into a single mix termed Mix 1, 2, or 3. Each mix was then enriched for phosphopeptides using TiO2. LC-MS/MS resolved the enriched phosphopeptides. This approach allows for direct comparison of phosphopeptide intensities within a mix consisting of light, medium, heavy isotope tags. **B** Phosphopeptide intensities between Coculture+Api in comparison with KG1a+Api were compared to depict upregulated phosphopeptides in increasing red hues and downregulated phosphopeptides in increasing blue hues. A scatter plot depicts log2-transformed values of phosphopeptides (*n* = 2381). Phosphosites having a missing value (no expression or undetected) were given a minimum intensity value for determining differential phosphorylation (log_2_-transformed LFQ values). **C** The proteome of KG1a Mono, HS-5 Mono, and KG1a-HS-5 Cocu label-free was determined in parallel to cross-validate enriched phosphopeptides. The top 1% phosphopeptide fold change (Coculture+Api minus KG1a+Api) is depicted by gene name and phosphopeptide fold change (blue). The corresponding protein abundance is plotted with the phosphopeptide in the stacked bars (gray). The fold changes are relative to KG1a+Api. **D** Differentially phosphorylated phosphosites with at least log2-transformed difference of 3 (309 phosphosites) compared with monoculture KG1a+Api were uploaded to ClueGo to determine enriched functionally grouped biological networks, *P* ≤ 0.05. The node size of the biological theme correlates with increasing significance. Functionally related groups overlap with color. For clarity, a subset pathway is depicted with gene name (circle indicates hyperphosorylation and triangle indicates hypophosphoryation) demonstrating the possible signal transduction of glycolysis proteins (red nodes) linking with AMPK signaling (blue nodes) in response to stroma-dependent protection of leukemia. This network highlights regulated genes mapped to the significantly enriched phosphosites, including ACSS2 (S30) and ACACA (S80). The complete biological pathways (≤0.05 *P* Value) of enriched phosphopeptides impacted by stroma-dependent protection of leukemia are depicted in Table 2.

The marked differences between HDACi sensitivity in leukemia monocultures compared to cocultures led us to investigate signaling mechanisms associated with cellular regulation through phosphorylation events. To this end, we applied mass spectrometry (MS)-based discovery phosphoproteomics to define the earliest leukemia-stroma activated pathways under protective coculture conditions. We treated leukemia monoculture and leukemia-stroma coculture with HDACi for 5 h, a pre-apoptotic phase with apicidin, (termed KG1a+Api and Coculture+Api, respectively), and used untreated monocultures and untreated coculture as reference signals. Multiplex isotope labeled lysates coupled with LC-MS/MS analysis was used to quantify PTMs of leukemia-stroma cocultures in comparison with monoculture cells including HDACi treated and untreated (Fig. 3A and Table 1). Relative quantification was only compared within the light, medium, and heavy signals and cross-validated across three independent labeling mixes (Mix 1, 2, or 3, isotope label scheme described in Table 1). The multiplex isotope labeling approach identified 2381 unique phosphosites from 1319 proteins with 684 phosphosites exceeding a log_2_-fold change of ±2 (Fig. 3B and Table 1). In order to determine the differentially phosphorylated sites leading to stroma-dependent protection of leukemic cells, relative quantification of phosphosite intensities of KG1a+Api (baseline) were subtracted from Coculture+Api (Mix 2). To identify highly relevant phosphosites we then surveyed the top 1% differentially phosphorylated sites (log_2_-fold change higher than >5 or lower than <5, Fig. 3C). Interestingly, two highly ranked phosphosites involving acetylation metabolism were identified: acyl-CoA synthetase 2 (ACSS2), which was up-regulated at phosphosite S30 (fold change: 6), and acetyl-CoA carboxylase alpha (ACACA), which was down-regulated at phosphosite S80 (fold change: −6.8) relative to KG1a+Api. Cross-validation in Mix 1 of ACSS2 (S30) indicated a >2-fold increase in intensity compared with Cocu+Api and Cocu relative to HS-5 Mono. Protein blot using phospho-ACACA (S80) antibody supports in validating these results in a decrease ACACA (S80) expression in Cocu (±Api) relative to KG1a and KG1a+Api (Supplementary Fig. 3A, B). Enzymatically, ACSS2 mediates cytosolic acetyl-CoA production from acetate and coenzyme A and the phosphorylation of S30 affects insulin signaling [38], whereas acetyl-CoA carboxylase alpha (ACACA) catalyzes the carboxylation of acetyl-CoA to malonyl-CoA in fatty acid synthesis [39] and its function is suppressed by phosphorylation of S80 in mice [40].

To assess how the phosphorylation profiles were associated with protein abundance, we measured and compared protein abundance in KG1a monoculture, HS-5 monoculture, and KG1a-HS-5 coculture. Relative quantification of protein abundance was determined by subtracting KG1a protein abundance intensities (baseline) from KG1a-Hs5. Thus, we uncovered 5.8-fold higher ACSS2 protein levels in coculture relative to KG1a monoculture having no detection value and were indeed in agreement with phosphorylation levels (Fig. 3C). Therefore, it is likely that up-regulation of ACSS2 (S30) in coculture stems from within the stroma cellular compartment. In a similar vein, ACACA protein levels were 2.3-fold lower in coculture compared to KG1a monoculture, mirroring the decrease in phosphorylation levels (Fig. 3C).

To gain a broader understanding of biological processes and pathways, we performed enrichment analyses of 3-fold differentially up- and down-regulated phosphoproteins using ClueGo with KEGG, Reactome, and gene ontology (GO) categories as resource databases. The most affected networks and pathways were PKB-dependent events, PI3K, mTOR signaling, glycolysis, EGF/EGFR, and AMP-activated protein kinase (AMPK) signaling (*P* ≤ 0.03, Table 2 and Fig. 3D, highlighted AMPK/mTOR signaling). Key phosphoproteins in AMPK/PI3K/mTOR signaling were stimulated by stroma-dependent protection with HDACi treatment of leukemia for which its protein network kinase activities have been previously shown to protect leukemia cells by suppressing oxidative stress in the bone marrow microenvironment [41]. Taken together, phosphorylation events leading to induce stroma-dependent protection of leukemia were identified by modulation with HDACi treatment.

**Table 2:** Significantly enriched biological pathways of top differentially phosphorylated phosphosites determined by Cytoscape ClueGo with Reactome, KEGG, and WikiPathways as ontology sources of stroma-dependent protection of leukemia cells.

GO Term	Ontology Source	Bonferroni *P*-Value	% Associated Genes	Nr. Genes	Genes in cluster
PKB-mediated events	REACTOME	0.01	9.72	7	EIF4B, IRS2, RPS6, TSC2, EIF4EBP1, EIF4G1, RPTOR
PI3K Cascade	REACTOME	0.01	9.72	7	EIF4B, IRS2, RPS6, TSC2, EIF4EBP1, EIF4G1, RPTOR
mTOR signaling	REACTOME	0.01	9.72	7	EIF4B, IRS2, RPS6, TSC2, EIF4EBP1, EIF4G1, RPTOR
mTORC1-mediated signaling	REACTOME	0.01	9.72	7	EIF4B, IRS2, RPS6, TSC2, EIF4EBP1, EIF4G1, RPTOR
Phosphorylation and inactivation of eEF2K by activated S6K1	REACTOME	0.01	9.72	7	EIF4B, IRS2, RPS6, TSC2, EIF4EBP1, EIF4G1, RPTOR
Glycolysis	REACTOME	0.01	16.67	5	PGAM1, TPI1, ENO1, GAPDH, PGK1
D-glyceraldehyde 3-phosphate + orthophosphate + NAD + < = > 1,3-bisphospho-D-glycerate + NADH + H+	REACTOME	0.01	16.67	5	PGAM1, TPI1, ENO1, GAPDH, PGK1
1,3-bisphospho-D-glycerate + NADH + H + < = > D-glyceraldehyde 3-phosphate + Orthophosphate + NAD+	REACTOME	0.01	16.67	5	PGAM1, TPI1, ENO1, GAPDH, PGK1
Insulin Signaling	WikiPathways	0.01	6.21	10	IRS2, MAP3K2, MAPK1, RRAD, TSC2, CAP1, EIF4EBP1, PRKCB, PRKCQ, TBC1D4
EGF/EGFR Signaling Pathway	WikiPathways	0.01	6.10	10	ATXN2, EPN1, ERRFI1, MAP3K2, MAPK1, MEF2D, RIN1, STMN1, EIF4EBP1, PRKCB
Interferon type I signaling pathways	WikiPathways	0.01	10.91	6	EIF4B, IRS2, RPS6, TYK2, EIF4EBP1, RPTOR
Brain-Derived Neurotrophic Factor (BDNF) signaling pathway	WikiPathways	0.01	6.25	9	BAD, IRS2, MAP3K2, MAPK1, MARCKS, RPS6, TSC2, EEF2, EIF4EBP1
Integrated Breast Cancer Pathway	WikiPathways	0.02	5.84	9	BAD, MAPK1, SMARCA4, TPR, TSC2, CDK7, CHEK1, MYC, PPP4R3A
AMPK signaling pathway	KEGG	0.02	6.61	8	CAMKK2, IRS2, TSC2, ACACA, EEF2, EIF4EBP1, ELAVL1, RPTOR
Glycolysis/Gluconeogenesis	KEGG	0.03	8.96	6	ACSS2, PGAM1, TPI1, ENO1, GAPDH, PGK1

### CRISPR-edited ACSS2 knockout in stroma modulates leukemia in an ACSS2-dependent manner

The phosphoproteome screen indicated that the acetyl-CoA processing enzymes ACSS2 and ACACA might be metabolically supporting leukemia through stroma interactions. Due to the up-regulation of the ACSS2 (S30) signal from cocultured stroma cells, we wanted to address the ACSS2 functional role of in a stroma-dependent manner. An antibody to follow ACSS2 (S30) phosphorylation was not commercially available therefore another approach was taken, single-guide RNAs (sgRNAs) with CRISPR/Cas9 gene editing were designed to target the phosphosite S30 of ACSS2 which impacted *ACSS2* expression in HS-5 stroma cells (termed HS-5 ACSS2-KO, Supplementary Fig. 4A). Surprisingly, HS-5 ACSS2-KO cells had a greater proliferative potential relative to wild type cells implicating a loss of ACSS2 could change proliferative rates in non-cancer cells (Supplementary Fig. 4B).

To test whether the loss of ACSS2 in stroma cells (HS-5 ACSS2-KO) impacted the viability of leukemic cells, we first examined apoptosis levels by treating leukemia cocultures with histone deacetylase inhibitors (Api, CBHA, and SAHA), with only API and SAHA treatment resulting in partial restoration of apoptosis in KG1a cocultured with HS-5 ACSS2-WT (Supplementary Fig. 4C). This result indicated that compensating metabolic pathways in HS-5 ACSS2-KO cells contributing to this path of proliferative growth and resistance may be involved [42].

**Fig. 4:**
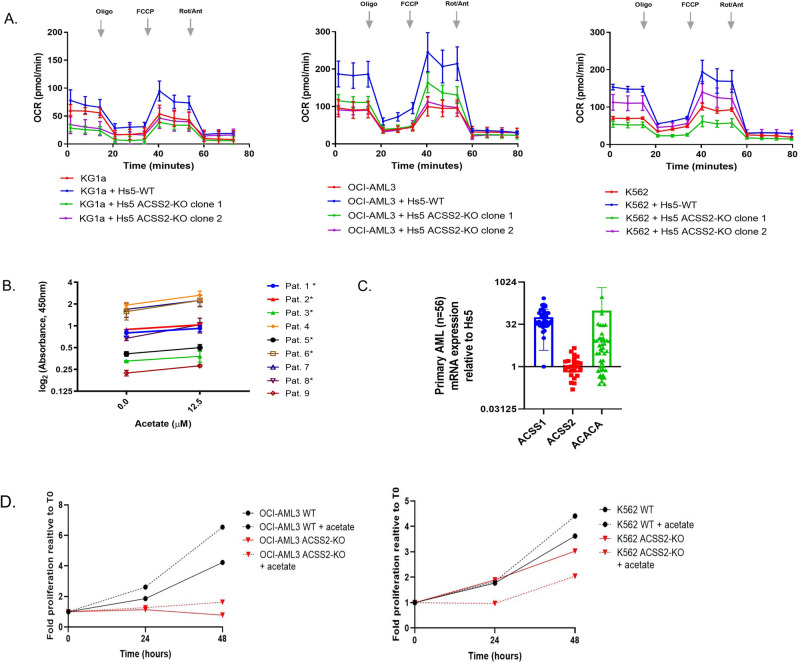
ACSS2 and metabolic substrate acetate modulate leukemic proliferation and metabolic fitness. **A** Seahorse XF Cell Mito Stress assay was used for assessing mitochondrial respiration of leukemia cells cultured overnight with stroma cells that were CRISPR-edited for ACSS2 (HS-5 ACSS2-KO) and compared with HS-5 WT. Leukemia cells (KG1a, OCI-AML3, or K562), monocultures, and cocultures were gently removed, equilibrated with modified Seahorse XF Cell Mito Stress RPMI buffer in a non-CO_2_ chamber at 37 °C, and re-platted into the provided manufacturer’s microwell plate to measure mitochondrial respiration OCAR output with Seahorse XFe96 instrument. Leukemia monoculture (red), leukemia pre-cultured with HS-5 (blue), and leukemia pre-cultured with HS-5 ACSS2-KO (clone 1, green or clone 2, purple) OCR is displayed at 5 min time points (mean ± SEM). Key parameters of oxidative respiration (mitochondrial respiration) are displayed as changes in rate mode at basal respiration, ATP-linked production, maximal respiration, and spare respiratory capacity by sequential injections of Oligo, FCCP, Rot/Ant. **B** Exogenous acetate treatment improved viability in primary AML blast specimens significantly in 6 of 9 specimens (paired t-test, *P* ≤ 0.05, indicated by an asterisk). **C** mRNA expression of ACSS1, ACSS2, and ACACA was measured in AML blasts (*n* = 46, solid circles). Measured in triplicate by standard SYBR Green quantitative RT PCR, plotted values display the relative mRNA expression measured relative to GAPDH and HS-5 (positive control). **D** Proliferative growth of OCI-AML3 WT and OCI-AML3 ACSS2-KO monocultures treated with acetate at 12.5 μM and measured at 0, 24, and 48 h (left plot). K562 WT and K562 ACSS2-KO were treated as OCI-AML3 (right plot). A solid black line depicts the proliferative growth of untreated WT cells while the black dashed line depicts the proliferative growth with acetate treatment. The blue lines depict the proliferative growth of OCI-AML3 ACSS2-KO cells untreated (solid line) and acetate treated (dashed). The red lines depict the proliferative growth of K562 ACSS2-KO cells untreated (solid line) and acetate treated (dashed).

ACSS2 is known to be important for tumor metabolism in hypoxic environments [43–45]. Likewise, in this study, AML-stroma proteomic analysis indicated upregulation of key metabolic pathways. We then wanted to examine the potential gain of metabolic function of leukemic cells gained from stroma contact including the role of ACCS2. In order to measure metabolic potential, OCR of leukemic cells was measured live in real-time. Leukemic cells were pre-exposed to HS-5 ACSS2-WT or to HS-5 ACSS2-KO overnight to allow cell-cell contacts then leukemic cells were gently removed, re-seeded in RPMI assay media for recalibration. Any contaminating stroma cells re-adhered to plastic and then only suspended leukemic cells were transferred to an assay plate at equal cell densities and subjected to hypoxia for 45 min for downstream mitochondrial respiration measurements. Leukemic monocultures were cultured in parallel as a control. The saturation oxygen levels (maximal OCR) were compared to the basal OCR to distinguish differences in mitochondrial respiration in leukemic cultures. Apparent steady rate increases of OCR in KG1a (2-fold), OCI-AML3 (1.5-fold) and K562 (1.5-fold) pre-exposed to HS-5 ACSS2-WT relative to monocultures were observed (Fig. 4A). In contrast, KG1a, OCI-AML3 and K562 pre-exposed to HS-5 ACSS2-KO had a markedly diminished OCR (2.7-fold, 1.7-fold, and 1.5-fold, respectively, Supplementary Fig. 4D) compared to leukemia cells pre-cultured with HS-5 ACSS2-WT. The results from these analyses indicate that leukemic cells pre-exposed to wild-type stroma gain metabolic respiratory capacity in a hypoxic environment dependent on ACSS2.

### The ACSS2 substrate acetate increases cell viability in leukemia

Extracellular acetate, a precursor of acetyl-CoA, has also been shown to contribute substantially to the increase in acetyl-CoA concentrations under hypoxic conditions through ACSS2 activity [45–48]. Therefore, we subjected cultured leukemic cell lines (KG1a, OCI-AML3, and K562) to an increasing concentration of acetate (0.5–5 μM) for 48 h to determine if leukemic proliferative growth was affected. The impact of acetate treatment on proliferative growth was 1.2- to 1.6-fold greater than on untreated leukemic cells (Supplementary Fig. 4E). To further validate this finding, we cultured primary AML blasts (n = 9) with exogenous acetate (12.5 µM; 48 h), which also resulted in a proliferative growth advantage of 1.2–1.4-fold in comparison to untreated AML blasts (Fig. 4B).

From the proteomic screen, we uncovered that KG1a ACSS2 protein abundance was undetected (confirmed by RT PCR), although acetate treatment enhanced proliferative growth in monocultures. We reasoned that *ACSS1*, an important paralog of *ACSS2*, would function as an alternate gene in processing acetate into acetyl-CoA in the absence of *ACSS2* [44, 49]. Therefore, we probed *ACSS1* mRNA expression in primary AML and, indeed, found that 32 of the 56 specimens expressed *ACSS1* at a staggering ~log2 fold change of 32 higher than *ACSS2* which was expressed in 8 of 56 specimens (Fig. 4C). Half of the cohort expressed elevated levels of *ACACA* relative to HS-5. Thus, surveying primary AML *ACSS1* and *ACSS2* expression profiles and the effects of exogenous acetate enhancement on proliferative growth yields the possibility that leukemic cells may uptake acetate from exogenous sources such as the microenvironment and process the metabolite through ACSS1or ACSS2.

Given that HDAC inhibitors were used to model drug resistance of AML, we examined acetylation profile of KG1a cells with histone mark H3K9, a post-translation modification associated with active transcription, to ask whether HDACi or acetate treatment transmits epigenetic modulation. By flow cytometry, we treated KG1a with HDACi (Api, 1 µM) for 48 h and measured intracellular levels of histone protein H3 and acetyl-modified histone residue, H3K9. We found that HDACi-treated KG1a cells resulted in increased expression of H3 and H3K9ac relative to unstained cells (Supplementary Fig. 5A, B). KG1a cells were also treated with acetate (48 h, 10 µM) resulted in a similar pattern of increased H3 and H3K9ac implicating that leukemic cell exposure to HDACi or exogenous acetate may play a vital role in the overall histone acetylation landscape of leukemic cells as previously shown in other cancer types [50, 51].

### Leukemic proliferative growth stimulated by exogenous acetate is dependent upon ACSS2

To test whether acetate uptake in leukemic cells was dependent on ACSS2 expression, ACSS2 single-guide RNAs (sgRNAs) were again utilized to knockout *ACSS2* expression in two leukemia cell lines expressing ACSS2, OCI-AML3, and K562. Monocultures of leukemic wild-type (WT) cells treated with exogenous acetate elicited a proliferative growth advantage compared to untreated cells, however proliferation was significantly diminished in ACCS2-KO leukemic cells (Fig. 4D). Our data support the view ACSS2 expression in leukemia is aimed at processing exogenous acetate to support proliferative growth.

### High expression of ACSS1/ACSS2 predicts poor overall survival in AML

**Fig. 5:**
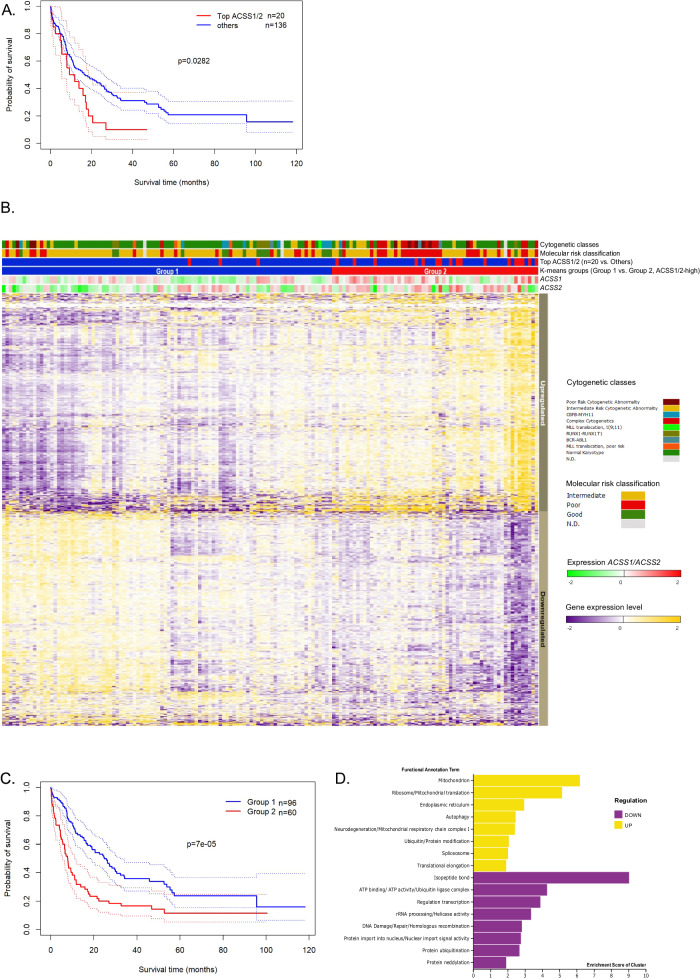
ACSS2 or ACSS1 high expression in the AML TCGA cohort predicts poor prognosis and relates to a distinct metabolic signature. **A** Kaplan-Meier estimation of the overall survival shows the poor outcome of patients in the top ACSS1/2 expression (red line, *n* = 20) compared to others (blue line, *n* = 136, log-rank test). Dotted lines mark the 95% confidence bounds. **B** Heatmap representation of differentially expressed genes in the TCGA AML dataset. Patients were plotted in the columns with the differential expressed genes in the rows. Top 20 *ACSS1*/*ACSS2* expression samples (*n* = 20 patients) were plotted in the horizontal rows above the heat map. Groups 1 and 2 show the division of the TCGA cohort into two groups using unsupervised clustering of this gene expression matrix (K-means for 2 groups) resulting in 1226 differentially expressed genes. Genes were classified as high or low according to the average expression of a gene in Group 2. **C** Kaplan-Meier estimation of the overall survival shows the poor outcome of patients in Group 2 (red line) compared to Group 1 (blue line, log-rank test). Dotted lines mark the 95% confidence bounds. **D** Functional annotation and enrichment cluster score from DAVID Bioinformatic Resources of genes up and downregulated with ACSS1/2 high expression are plotted.

We further explored the clinical impact of *ACSS1, ACSS2*, and *ACACA* expression in AML in terms of overall survival. We reasoned that the expression of *ACSS1* and *ACSS2* would carry clinical relevance based on biochemical function. Therefore, we examined the mRNA expression of *ACSS1* and *ACSS2* using AML TCGA RNA-seq dataset [30] that included different cytogenetic AML subtypes (*n* = 156). We defined two groups of AML samples using patients with highest gene expression values of *ACSS1* (*n* = 10) and highest gene expression values of *ACSS2* (*n* = 10) denoted as top ACSS1/2 samples (*n* = 20). These cases displayed inferior outcome survival (OS) probability compared to the remaining samples (top ACSS1/2 median OS was 9.2 months versus Others with median 16.4 months; *p* = 0.028, Fig. 5A). We found 1225 genes differentially expressed between these two groups (Fig. 5B). Using K-means clustering to statistically partition these expression patterns into two groups matching the expression pattern of 1225 differentially expressed genes, we obtained a broader group of patients with ACSS1/2-high expression levels (Group 2, *n* = 60) and with ACSS1/2-low expression levels (Group 1, *n* = 96, Fig. 5B). K-means clustering remained statistically significant in further partitioning into 4 groups. Interestingly, the two-group cluster also displayed different survival probabilities with an inferior outcome for patients in ACSS1/2-high (Group 2, median OS of 7.8 months versus Group 1 median OS of 24.8 months, *p* = 7E-05, Fig. 5C). The up-regulated genes include enrichment of 83 (14%) mitochondrial proteins (Fig. 5D and listed in Supplementary Dataset 5). ACSS1/2-high patients were significantly older and encompassed almost all the molecular subtypes, and significantly harbored high molecular risk AML cases including TP53 mutated AML. ACSS1/2-high was of prognostic significance in a uni- and multivariate analysis (Supplementary Table 2A, B) and remains significant when including only intensively treated patients in the ACSS1/2-high group (*p* = 0.042).

## Discussion

Using an unbiased discovery phosphoproteomics approach, we identify ACSS2, as a potential mechanism in protecting leukemic cells to survive therapies through a combined metabolic-based response. We reached this conclusion by performing stroma-dependent co-culture protection modeling with HDACi treatment, implicating that acetate is involved in the resistance of leukemia. Importantly, the conclusion is supported by a metabolic-based response of AML-stroma co-cultures, up-regulation of acetate-processing enzyme ACSS2 (S30), acetate-enhanced leukemic growth with ACSS2 dependence, abolished leukemic metabolic fitness with ACSS2 mutant stroma and analyses of *ACSS1/ACSS2*-high mRNA expression in AML datasets.

Studies reporting leukemia dependence on the bone marrow microenvironment have provided mechanistic clues on the resistance to therapy [4, 6, 7, 9, 10] and conversely, we previously reported that AML pathology is imprinted on the aberrant gene expression patterns and high mutational frequencies in AML-derived stroma [13]. In this study, phosphoproteomic profiling of leukemia-stroma interaction provides phosphorylation sites of proteins invoked by the cell-to-cell communication paths. Phosphoproteins enriched for glycolysis/catabolic processes and AMPK/mTOR signaling pathway, signaling drew our attention to the signaling paths of up- and down-regulated proteins, ACSS2 (S30) and ACACA (S80) both involved in de novo lipogenesis [50, 51] and both of which are regulated through AMPK/mTOR [49]. Moreover, the compelling evidence on the role of ACSS2 in regulating the acetate levels through nuclear histone acetylation, fatty acids biosynthesis, and recycling of acyl groups when glucose or oxygen is deprived in cancer led us to further explore the role of ACSS2 in AML [44–47, 51].

Evidence as to how acetate becomes available is just emerging [52–54] but if available, acetate in the form of acetyl coA can be used in energy production, lipid synthesis, and protein acetylation including histone acetylation [51] and possibly affecting the epigenetic response in AML [24]. We highlight herein that HDACi, Class I HDACs, promoted stroma-dependent protection possibly affecting nuclear acetate levels to support drug resistance in leukemia, a mechanism mirrored in bacterial acetyl coA synthetase (acs) by switching to acetate in developing antibiotic resistance [55]. We also show that acetate treatment additionally invokes a leukemic ACSS2-dependent proliferative growth advantage, implicating uptake and conversion into acetyl-CoA. Cancer growth dependence on ACSS2 has been previously demonstrated in tumors where reduced tumor burden was shown in ACSS2^-/-^ and shACSS2 models [45, 47]. Interestingly KG1a treated with acetate, although lacking ACSS2 expression, also resulted in enhanced proliferative growth. Through this serendipitous result, the expression of an alternate ACSS2 paralog, *ACSS1* was uncovered as a potential factor in AML. It has been reported that both ACSS1 and ACSS2 can produce acetyl-CoA from acetate but in distinct cellular compartments, mitochondria, and nucleo-cytosol, respectively, indicating that this distinction may partition the type of metabolic response [49, 54]. Also, *ACSS1* and *ACSS2* expression levels can affect the net acetate uptake rates in several cancer types [48].

Our study also introduces an additional role for ACSS2 (S30) within stroma where we asked whether ACSS2 influenced mitochondrial respiration of leukemic cells. Our findings show that leukemia pre-exposure to HS-5 WT enhanced OCR compared to leukemia monocultures (KG1a, OCI-AML3, and K562) but pre-exposure to HS-5 ACSS2-KO abolished this observed enhanced metabolic or “respiratory” state. Recent studies describe increased acetate secretion and its incorporation into TCA and fatty acid synthesis in leukemia cocultures [53]. The results corroborate with our findings by a different approach that leukemia cells gain metabolic fitness. In addition, we also attempted to override stroma-dependent protection of leukemia through cocultures with HS-5 ACSS2-KO and HDACi treatment however we only observed partially restored apoptosis levels indicating that anti-apoptotic compensating regulatory metabolic paths were likely involved and should also be further explored by metabolomics studies. ACSS2 inhibitors currently in Phase I study for metastatic tumors (NCT04990739) or a transition state ACSS2 mimetic [53, 56] may be better suited for a full apoptotic restoration to prevent stroma-dependent protection.

In addition to the specific role of ACSS2, we identified here, an enhanced respiratory state by AML-stroma interactions was also captured by a broader metabolic-based proteomic response consisting of proteins involved in glycolysis, mitochondrial processes, and fatty acid synthesis that also included translation biosynthesis, protease complex, nuclear import signaling. Interestingly, NAMPT, the most upregulated of the metabolic proteins, replenishes NAD^+^ levels and is a cofactor required for glycolysis, oxidative phosphorylation, and fatty acid synthesis [57] implicating a high energy demand signal from leukemia cells upon stroma contact.

Some major limitations of these this study remain such as the small AML cohort size that does not represent the heterogeneity of AML. Another limitation was the use of a single healthy stroma cell line (HS-5) giving a constant parameter of normal stroma for surveying different types of leukemia, however, phosphorylation changes may be cell-line specific. Healthy stroma and AML-derived stroma have been shown to differ in how leukemia is supported by kinetic growth and/or protected creating altered niches [8]; thus further studies are warranted to consider the dynamics of AML-derived stroma. Despite these variables, by modeling stroma protection of leukemia with HDACi, ACSS2 was identified as a pertinent protein in leukemia-stroma interactions and corroborates with previously reported studies on the metabolic effects of leukemia-stroma cellular interactions [58, 59].

Poor outcome associated with elevated ACSS2 expression has been reported in human triple-negative breast cancers, glioblastoma and brain metastases, non-small-cell lung carcinoma, cervical squamous cell carcinoma [47, 48, 60, 61], and in concordance, we similarly uncovered a poor outcome AML subtype featuring *ACSS1*/*ACSS2*-high, associated with TP53 mutations, and an enrichment of mitochondrion genes indicating a causal role for ACSS1/ACSS2. The *ACSS1*/*ACSS2*-high subtype of AML also coincides well with the recent proteogenomic characterization of AML with a mitochondrial gene-enriched phenotype [12].

Taken together, we identify the phosphoproteome signature leading to leukemic cell protection by an epigenetic-induced drug resistance path dependent on the interconnectivity with stroma including ACSS2 function. We also highlight the clinical relevance of *ACSS1/ACSS2*-high levels in AML. Elevated expression of *ACSS1/2* patients can serve as a targetable biomarker for patient stratification and may improve therapy response in *ACSS1/ACSS2*-high AML subtype.

## Supplementary information

Supplementary

Dataset 1 AML-Stroma Proteome.

Dataset 2 80 epigenetic compound description.

Dataset 3 Mix 1, 2, and 3 top hit candidate protein abundances and phosphosites.

Dataset 4 Proteome dataset: KG1a, HS-5, and coculture proteome.

Dataset 5 TCGA ACSS1/2-high.

## Data Availability

AML proteomic data generated in this study have been submitted to ProteomeXchange Consortium via the PRIDE partner repository with the dataset identifier PD032051. Processed phosphoproteomic data can be accessed at 10.6084/m9.figshare.23261309. All other datasets are under supplementary materials.

## References

[1] Dohner H, Weisdorf DJ, Bloomfield CD. Acute myeloid leukemia. N. Engl J Med. 2015;373:1136–52. 26376137 10.1056/NEJMra1406184

[2] Feldhahn N, Arutyunyan A, Stoddart S, Zhang B, Schmidhuber S, Yi S-J, et al. Environment-mediated drug resistance in Bcr/Abl-positive acute lymphoblastic leukemia. Oncoimmunology. 2012;1:618–29. 22934254 10.4161/onci.20249 PMC3429566

[3] Zahreddine H, Borden KLB. Mechanisms and insights into drug resistance in cancer. Front Pharm. 2013;4:1–8. 10.3389/fphar.2013.00028 PMC3596793 23504227

[4] Duarte D, Hawkins ED, Lo Celso C. The interplay of leukemia cells and the bone marrow microenvironment. Blood. 2018;131:1507–11. 29487069 10.1182/blood-2017-12-784132

[5] Papaccio F, Paino F, Regad T, Papaccio G, Desiderio V, Tirino V. Concise review: cancer cells, cancer stem cells, and mesenchymal stem cells: influence in cancer development. Stem Cells Transl Med. 2017;6:2115–25. 29072369 10.1002/sctm.17-0138 PMC5702541

[6] Olga Blau (2011). Bone Marrow Microenvironment in the Pathogenesis of AML, Myeloid Leukemia - Basic Mechanisms of Leukemogenesis, Dr Steffen Koschmieder (Ed.,185–96).

[7] Korn C, Mendez-Ferrer S. Myeloid malignancies and the microenvironment. Blood. 2018;129:811–23. 10.1182/blood-2016-09-670224 PMC5314811 28064238

[8] Ramasamy R, Lam EWF, Soeiro I, Tisato V, Bonnet D, Dazzi F. Mesenchymal stem cells inhibit proliferation and apoptosis of tumor cells: Impact on in vivo tumor growth. Leukemia. 2007;21:304–10. 17170725 10.1038/sj.leu.2404489

[9] Konopleva M, Konoplev S, Hu W, Zaritskey AY, Afanasiev BV, Andreeff M. Stromal cells prevent apoptosis of AML cells by up-regulation of anti-apoptotic proteins. Leukemia. 2002;16:1713–24. 12200686 10.1038/sj.leu.2402608

[10] Garrido SM, Appelbaum FR, Willman CL, Banker DE. Acute myeloid leukemia cells are protected from spontaneous and drug-induced apoptosis by direct contact with a human bone marrow stromal cell line (HS-5). Exp Hematol. 2001;29:448–57. 11301185 10.1016/S0301-472X(01)00612-9

[11] Elstrom RL, Bauer DE, Buzzai M, Karnauskas R, Harris MH, Plas DR, et al. Akt stimulates aerobic glycolysis in cancer cells. Cancer Res. 2004;64:3892–9. 15172999 10.1158/0008-5472.CAN-03-2904

[12] Vander Heiden MG, Cantley LC, Thompson CB. Understanding the Warburg effect: the metabolic requirements of cell proliferation. Science. 2009;324:1029–33. 19460998 10.1126/science.1160809 PMC2849637

[13] Heide, von der EK, Neumann M, Vosberg S, James AR, Schroeder MP, et al. Molecular alterations in bone marrow mesenchymal stromal cells derived from acute myeloid leukemia patients. Leukemia. 2017;31:1069–78. 27833093 10.1038/leu.2016.324

[14] Kornblau SM, Ruvolo PP, Wang RY, Battula VL, Shpall EJ, Ruvolo VR, et al. Distinct protein signatures of acute myeloid leukemia bone marrow-derived stromal cells are prognostic for patient survival. Haematologica. 2018;103:810–21. 29545342 10.3324/haematol.2017.172429 PMC5927978

[15] Aasebø E, Brenner AK, Birkeland E, Tvedt THA, Selheim F, Berven FS, et al. The constitutive extracellular protein release by acute myeloid leukemia cells—a proteomic study of patient heterogeneity and its modulation by mesenchymal stromal cells. Cancers. 2021;13:1509. 33806032 10.3390/cancers13071509 PMC8037744

[16] Aasebø E, Brenner AK, Hernandez-Valladares M, Birkeland E, Mjaavatten O, Reikvam H, et al. Patient heterogeneity in acute myeloid leukemia: leukemic cell communication by release of soluble mediators and its effects on mesenchymal stem cells. Diseases. 2021;9:74. 34698165 10.3390/diseases9040074 PMC8544451

[17] Huang JC, Basu SK, Zhao X, Chien S, Fang M, Oehler VG, et al. Mesenchymal stromal cells derived from acute myeloid leukemia bone marrow exhibit aberrant cytogenetics and cytokine elaboration. Blood Cancer J. 2015;5:e302–9. 25860293 10.1038/bcj.2015.17 PMC4450324

[18] Jacamo R, Chen Y, Wang Z, Wencai M, Zhang M, Spaeth EL, et al. Reciprocal leukemia-stroma VCAM-1/VLA-4-dependent activation of NF-κB mediates chemoresistance. Blood. 2014;123:2691–702. 24599548 10.1182/blood-2013-06-511527 PMC3999754

[19] Jayavelu AK, Wolf S, Buettner F, Alexe G, Häupl B, Comoglio F, et al. The proteogenomic subtypes of acute myeloid leukemia. Cancer Cell. 2022;140:1533–48. 10.1016/j.ccell.2022.02.006 PMC12882723 35245447

[20] Leung KK, Nguyen A, Shi T, Tang L, Ni X, Escoubet L, et al. Multiomics of azacitidine-treated AML cells reveals variable and convergent targets that remodel the cell-surface proteome. Proc Natl Acad Sci USA. 2019;116:695–700. 30584089 10.1073/pnas.1813666116 PMC6329958

[21] Kornblau SM, Tibes R, Qiu YH, Chen W, Kantarjian HM, Andreeff M, et al. Functional proteomic profiling of AML predicts response and survival. Blood. 2009;113:154–64. 18840713 10.1182/blood-2007-10-119438 PMC2951831

[22] Kramer MH, Zhang Q, Sprung R, Day RB, Erdmann-Gilmore P, Li Y, et al. Proteomic and phosphoproteomic landscapes of acute myeloid leukemia. Blood [Internet]. J Am Soc Hematol. 2022;140:1533–48. 10.1182/blood.2022016033 PMC9523374 35895896

[23] Kaelin WG, McKnight SL. Influence of metabolism on epigenetics and disease. Cell. 2013;153:56–69. 23540690 10.1016/j.cell.2013.03.004 PMC3775362

[24] Dhall A, Zee BM, Yan F, Blanco MA. Intersection of epigenetic and metabolic regulation of histone modifications in acute myeloid leukemia. Front Oncol. 2019;9:1–10. 31192132 10.3389/fonc.2019.00432 PMC6540842

[25] Abdel-Wahab O, Levine RL. Mutations in epigenetic modifiers in the pathogenesis and therapy of acute myeloid leukemia. Blood. 2013;121:3563–72. 23640996 10.1182/blood-2013-01-451781 PMC3643757

[26] Meldi KM, Figueroa ME. Epigenetic deregulation in myeloid malignancies. Transl Res. 2014;165:102–14. 24813528 10.1016/j.trsl.2014.04.012

[27] Rappsilber J, Mann M, Ishihama Y. Protocol for micro-purification, enrichment, pre-fractionation and storage of peptides for proteomics using StageTips. Nat Protoc. 2007;2:1896–906. 17703201 10.1038/nprot.2007.261

[28] Boersema PJ, Raijmakers R, Lemeer S, Mohammed S, Heck AJR. Multiplex peptide stable isotope dimethyl labeling for quantitative proteomics. Nat Protoc. 2009;4:484–94. 19300442 10.1038/nprot.2009.21

[29] Thingholm TE, Jørgensen TJD, Jensen ON, Larsen MR. Highly selective enrichment of phosphorylated peptides using titanium dioxide. Nat Protoc. 2006;1:1929–35. 17487178 10.1038/nprot.2006.185

[30] TCGA. Genomic and epigenomic landscapes of adult de novo acute myeloid leukemia. N. Engl J Med. 2013;368:2059–74. 23634996 10.1056/NEJMoa1301689 PMC3767041

[31] Perez-Llamas C, Lopez-Bigas N. Gitools: analysis and visualisation of genomic data using interactive heat-maps. PLoS One. 2011;6:e19541. 21602921 10.1371/journal.pone.0019541 PMC3094337

[32] Schroeder MP, Gonzalez-perez A, Lopez-bigas N. Visualizing multidimensional cancer genomics data. Genome Med. 2013;5:1–13. 23363777 10.1186/gm413 PMC3706894

[33] Yang M, Zhang L, Stevens J, Gibson G. CRISPR/Cas9 mediated generation of stable chondrocyte cell lines with targeted gene knockouts; analysis of an aggrecan knockout cell line. Bone Elsevier Inc 2014;69:118–25. 10.1016/j.bone.2014.09.005 25260929

[34] Sheng L, Mason C, Melnick A. Genetic and epigenetic heterogeneity in acute myeloid leukemia. Curr Opin Genet Dev. 2016;36:100–6. 27162099 10.1016/j.gde.2016.03.011 PMC4903929

[35] Goldman SL, Hassan C, Khunte M, Soldatenko A, Jong Y, Afshinnekoo E, et al. Epigenetic modifications in acute myeloid leukemia: prognosis, treatment, and heterogeneity. Front Genet. 2019;10:133. 30881380 10.3389/fgene.2019.00133 PMC6405641

[36] Rashkovan M, Ferrando A. Metabolic dependencies and vulnerabilities in leukemia. Genes Dev. 2019;33:1460–74. 31676734 10.1101/gad.326470.119 PMC6824464

[37] Ni Y, Zhou X, Yang J, Shi H, Li H, Zhao X, et al. The role of tumor-stroma interactions in drug resistance within tumor microenvironment. Front Cell Dev Biol. 2021;9:1–29. 10.3389/fcell.2021.637675 PMC8173135 34095111

[38] Asfa AS, Qiu B, Wee S, Choi H, Gunaratne J, Tergaonkar V. Phosphoprotein network analysis of white adipose tissues unveils deregulated pathways in response to high-fat diet. Sci Rep. 2016;6:25844. 27180971 10.1038/srep25844 PMC4867603

[39] Röhrig F, Schulze A. The multifaceted roles of fatty acid synthesis in cancer. Nat Rev Cancer. 2016;16:732–49. 27658529 10.1038/nrc.2016.89

[40] Lally JSV, Ghoshal S, DePeralta DK, Moaven O, Wei L, Masia R, et al. Inhibition of acetyl-CoA carboxylase by phosphorylation or the inhibitor ND-654 suppresses lipogenesis and hepatocellular carcinoma. Cell Metab. 2019;29:174–.e5. 30244972 10.1016/j.cmet.2018.08.020 PMC6643297

[41] Saito Y, Chapple RH, Lin A, Kitano A, Nakada D. AMPK protects leukemia-initiating cells in myeloid leukemias from metabolic stress in the bone marrow. Cell Stem Cell. 2015;17:585–96. 26440282 10.1016/j.stem.2015.08.019 PMC4597792

[42] Zhao S, Torres AM, Henry RA, Trefely S, Wallace M, Lee JV, et al. ATP-citrate lyase controls a glucose-to-acetate metabolic switch. Cell Rep. 2016;17:1037–52. 27760311 10.1016/j.celrep.2016.09.069 PMC5175409

[43] Kamphorst JJ, Chung MK, Fan J, Rabinowitz JD. Quantitative analysis of acetyl-CoA production in hypoxic cancer cells reveals substantial contribution from acetate. Cancer Metab. 2014;2:23. 25671109 10.1186/2049-3002-2-23 PMC4322440

[44] Bulusu V, Tumanov S, Michalopoulou E, van den Broek NJ, MacKay G, Nixon C, et al. Acetate recapturing by nuclear acetyl-CoA synthetase 2 prevents loss of histone acetylation during oxygen and serum limitation. Cell Rep. 2017;18:647–58. 28099844 10.1016/j.celrep.2016.12.055 PMC5276806

[45] Schug ZT, Peck B, Jones DT, Zhang Q, Grosskurth S, Alam IS, et al. Acetyl-CoA synthetase 2 promotes acetate utilization and maintains cancer cell growth under metabolic stress. Cancer Cell. 2015;27:57–71. 25584894 10.1016/j.ccell.2014.12.002 PMC4297291

[46] Noia D, Neuberger JM, Keim MS, Kazadi C, Rothschild D, Basu G, et al. Acetate fuels the cancer engine. Cell. 2014;159:1492–4. 25525870 10.1016/j.cell.2014.12.009

[47] Comerford SA, Huang Z, Du X, Wang Y, Cai L, Witkiewicz AK, et al. Acetate dependence of tumors. Cell. 2014;159:1591–602. 25525877 10.1016/j.cell.2014.11.020 PMC4272450

[48] Mashimo T, Pichumani K, Vemireddy V, Hatanpaa KJ, Singh DK, Sirasanagandla S, et al. Acetate is a bioenergetic substrate for human glioblastoma and brain metastases. Cell. 2014;159:1603–14. 25525878 10.1016/j.cell.2014.11.025 PMC4374602

[49] Sivanand S, Viney I, Wellen KE. Spatiotemporal control of acetyl-CoA metabolism in chromatin regulation. Trends Biochem Sci. 2018;43:61–74. 29174173 10.1016/j.tibs.2017.11.004 PMC5741483

[50] Li X, Yu W, Qian X, Xia Y, Zheng Y, Lee J, et al. Nucleus-translocated ACSS2 promotes the gene transcription for lysosomal biogenesis and autophagy. Mol Cell. 2017;66:684–97. 28552616 10.1016/j.molcel.2017.04.026 PMC5521213

[51] Gao, Lin X, Ren S-H, Li F, Chen J-JJ J-T, Yao C-B, et al. Acetate functions as an epigenetic metabolite to promote lipid synthesis under hypoxia. Nat Commun Nat Publ Group. 2016;7:1–14. 10.1038/ncomms11960 PMC4931325 27357947

[52] Liu X, Cooper DE, Cluntun AA, Warmoes MO, Zhao S, Reid MA, et al. Acetate production from glucose and coupling to mitochondrial metabolism in mammals. Cell. 2018;175:502–.e13. 30245009 10.1016/j.cell.2018.08.040 PMC6173642

[53] Vilaplana-Lopera N, Cuminetti V, Almaghrabi R, Papatzikas G, Rout AK, Jeeves M, et al. Crosstalk between AML and stromal cells triggers acetate secretion through the metabolic rewiring of stromal cells. Elife. 2022;11:1–29. 10.7554/eLife.75908 PMC9477493 36052997

[54] Jaworski DM, Namboodiri AMA, Moffett JR. Acetate as a metabolic and epigenetic modifier of cancer therapy. J Cell Biochem. 2016;117:574–88. 26251955 10.1002/jcb.25305

[55] Wolfe AJ. The acetate switch. Microbiol Mol Biol Rev. 2005;69:12–50. 15755952 10.1128/MMBR.69.1.12-50.2005 PMC1082793

[56] Miller KD, Pniewski K, Perry CE, Papp SB, Shaffer JD, Velasco-Silva JN, et al. Targeting ACSS2 with a transition-state mimetic inhibits triple-negative breast cancer growth. Cancer Res. 2021;81:1252–64. 33414169 10.1158/0008-5472.CAN-20-1847 PMC8026699

[57] Navas LE, Carnero A. NAD+ metabolism, stemness, the immune response, and cancer. Signal Transduct Target Ther. 2021;6:2. 33384409 10.1038/s41392-020-00354-w PMC7775471

[58] Marlein CR, Zaitseva L, Piddock RE, Robinson SD, Edwards DR, Shafat MS, et al. NADPH oxidase-2 derived superoxide drives mitochondrial transfer from bone marrow stromal cells to leukemic blasts. Blood. 2017;130:1649–60. 28733324 10.1182/blood-2017-03-772939

[59] Moschoi R, Nebout M, Chiche J, Mary D, Prebet T, Saland E, et al. Protective mitochondrial transfer from bone marrow stromal cells to acute myeloid leukemic cells during chemotherapy. Blood. 2016;128:253–65. 27257182 10.1182/blood-2015-07-655860

[60] Yang X, Shao F, Shi S, Feng X, Wang W, Wang Y, et al. Prognostic impact of metabolism reprogramming markers acetyl-CoA synthetase 2 phosphorylation and ketohexokinase-a expression in non-small-cell lung carcinoma. Front Oncol. 2019;9:1–8. 31750240 10.3389/fonc.2019.01123 PMC6848158

[61] Li CJ, Chiu YH, Chang C, Chang YCI, Sheu JJC, Chiang AJ. Acetyl coenzyme a synthase 2 acts as a prognostic biomarker associated with immune infiltration in cervical squamous cell carcinoma. Cancers. 2021;13:3125. 34206705 10.3390/cancers13133125 PMC8269092

