# Supplementary material for: Proteomic profiling reveals ACSS2 facilitating metabolic support in acute myeloid leukemia
Source: Cancer Gene Ther. 2024 Jun 8;31(9):1344–56. doi: 10.1038/s41417-024-00785-5 (PMC11405269; doi:10.1038/s41417-024-00785-5)
Supplement: Supplementary file 1 — Supplementary [file 41417_2024_785_MOESM1_ESM.pdf]

Supplement Materials and Methods

LC-MS analysis for shotgun proteomics

Five microliters of each sample were injected in duplicates on a LC-MS/MS system NanoLC 400 (Eksigent) coupled to Q Exactive Plus (Thermo, only cell line samples) or a Q Exactive HF (Thermo only AML samples), using a 240 min gradient ranging from 5% to 40% of solvent B (80% acetonitrile, 0.1 % formic acid; solvent A= 5 % acetonitrile, 0.1 % formic acid). For the chromatographic separation 100 cm-long MonoCap C18 HighResolution 2000 (GL Sciences) was used.

The nanospray source was operated with a spay voltage of 2.3 kV and an ion transfer tube temperature of 260°C. Data were acquired in data-dependent mode, with a top10 method. On the Q Exactive Plus, one survey MS scan with a resolution of 70,000 at m/z 200 was used, followed by up to 10 MS/MS scans on the most intense ions, with a resolution of 17,500 and intensity threshold of 5,000). On the Q Exactive HF one survey MS scan with a resolution of 120,000 at m/z 200 was used, followed by up to 10 MS/MS scans on the most intense ions, with a resolution of 30,000 and intensity threshold of 5,000. Once selected for fragmentation, ions were excluded from further selection for 45 sec to increase new sequencing events. Raw data were analyzed using the MaxQuant proteomics pipeline (v1.5.3.30 or 1.4.1.2) and the built-in Andromeda search engine (24) with the human Uniprot database. Carbamidomethylation of cysteines was chosen as a fixed modification whereas oxidation of methionine and acetylation of N-terminus were chosen as variable modifications. The search engine peptide assignments were filtered at 1% FDR and the feature match between runs was enabled; other

parameters were left as default. Data quality was inspected using the in-house developed tool PTXQC (25). A correlation plot indicated a low variation of protein expression across all quality control samples, thus no normalization was required before data analysis. A total of 6754 proteins were quantitated. A filter was applied to retain proteins having LFQ intensity values in at least 14 of the 28 samples, yielding a total of 2231 unique proteins. Differential protein expression between AML Mono and AML Cocu was determined using the limma package in the statistical language R, requiring a fold change ≥1.5 and <0.5, q-value ≤0.05.

Mass Spectrometry and data preprocessing for phosphoproteomics of KG1a, Hs5, and KG1a-Hs5

Samples were analyzed using a QExactive mass spectrometer (Thermo Scientific) and a U3000 RSLC nano HPLC system (Dionex) coupled to a 50 cm-long and 75-micron internal diameter EASYspray column. Samples were resolved using a 4 h gradient with the mass spectrometer run in data-dependent analysis mode, in which the 20 most intense multiply charged precursors were selected for fragmentation. The mass spectrometer settings were as follows: Precursor resolution; 70000, AGC target; 3000000, maximum fill time; 250 ms, MSMS resolution; 17500, AGC target 100000' maximum fill time was 120 ms, isolation window; 3 Da, normalized collision energy (NCE); 30, underfill ratio 10%. Data were processed and quantified using Proteome Discoverer 1.4 (Thermo Scientific) and Mascot (MatrixScience). Search parameters in Mascot (via PD) were as follows – Enzyme (trypsin), Dynamic modifications: Oxidation (M), Phosphorylation (STY), Dimethyl light, intermediate or heavy of (K) and (N-term); Static

modifications: Carbamidomethyl (C); Precursor Mass tolerance: 10 ppm; Fragment Mass tolerance 0.02 Da; Missed Cleavages: 2. Data were searched against the UniProt Homo sapiens reference proteome version 2.5 (retrieved on 08/12/2013). Site localization probabilities were calculated by PhosphoRS 3.154, which is implemented in Proteome Discoverer. The statistical programming language R (https://www.r-project.org) was used for subsequent data analysis and visualization. Peptide data were extracted from Proteome Discoverer and analyzed using an in-house R script. Peptide-spectrum matches were filtered at a 1% False Discovery Rate (sequence identification); the list of phosphorylated peptides was then further filtered to have a site localization score of at least 0.75. Phosphosite abundances were transformed to log2 scale and normalized to mean 0 and standard deviation 2 separately for each time point and each measurement. Processed phosphoproteomic data can be accessed at 10.6084/m9.figshare.23261309.

Statistical analysis of AML datasets

AML RNA expression dataset from TCGA (29) was used to determine the impact of metabolic gene expression in AML. The TCGA RNA-seq dataset was pre-processed by Pancan12 (24), which is a log2-RSEM transformed and median-centered divided by standard deviation of 17262 genes. This dataset, available as a Gitools 2.3.1 matrix (32, 33), was used to examine differential gene expression for 156 samples (excluding FAB M3 samples). Group comparisons were analysed with a Mann-Whitney-Wilcoxon test and considered significant when P-value was

<0.01. Unsupervised clustering of samples was performed by K-means using Euclidean distance (K=2 and 30k iterations). Correlations between continuous factors of specified groups of patients were calculated with Kruskal-Wallis rank sum test, while dichotomous factors were tested with Pearson's chi-squared, both using the ‘tableBy’ function in the ‘arsenal’ package in R.

To study the impact of the metabolic gene expression on patient survival, the overall survival (in months, mo) was performed using the R package ‘survival’, with times censored at the last follow-up. Differences between groups were tested with unadjusted Kaplan-Meier curves using log-rank tests. Hazard ratios were estimated from Cox proportional hazard regression and used to evaluate the independent effects of covariates. Univariate and multivariate analyses were evaluated for Cox proportionality. The enrichments were confirmed using DAVID 6.8 from which only enrichment clusters with P-value≥.01 were considered.

Supplement Legends

Supplement Fig. S1A and B. Leukemia cell viability was measured by treatment with an 80-compound library targeting epigenetic protein families. The proliferative growth of 9 leukemia cell lines encompassing both acute myeloid and lymphoblastic leukemia diseases was measured. The effectiveness of each compound was measured at 1µM (left) and 10µM (right) concentrations for 48h. Purple hues (ratio to untreated >1.25) indicate proliferative growth advantage and red hues indicate diminished proliferative growth (< 0.75) and a ratio of 0.76 to 1.24 indicate no effect. The compound library was clustered and color coded according to the targeted function of the epigenetic protein family ie, histone deacetylase inhibitors (HDACi), histone methyltransferases (HTMi), bromodomain (BETi), DNA methylase (DNMTi), sirtuins (SIRTi), and other. Compound name, drug ID (G4, B5, etc.), and color code are listed in the columns right of the drug class. Each proliferative measurement was conducted in 6 measurements, which were averaged. Ratios were calculated as treated/untreated with the averaged O.D. 460nm measurement.

Supplement Fig. S2. Stroma-dependent protection of primary AML depicted in three diagrams A. Percent apoptotic response of AML Mono and AML Cocu treated with HDACi (C11, C3, A8, B8, and H7) for 48 h at 10µM concentration. The hues represent the HDACi compound. B. The significance apoptotic difference of AML Mono versus AML Cocu as determined by paired student t-test (symbols *, **, and *** indicate P-value < 0.05, < 0.01, < 0.001, respectively). C. AML ID with

unique hue colors to distinguish the AML samples. D. Percent apoptotic response of OCI-AML3 and K562 cell lines treated with HDACi (C11, C3, A8, B8, and H7) for 48 h at 10μM concentration. All values in a paired t-test between monoculture and coculture were significant (p-value <0.001) for HDACi tested.

Supplement Fig. S3A left and right, A, Quantitation of phosphosites in ACSS2 S30 and ACACA S80 detected in both Mix1 and Mix2. Values are normalized and plotted as Log2 values. Light (L), Medium (M), and Heavy (H) refers to the isotope label. **B,** Western blot depicts representative protein levels of ACACA (Ser79) and β-actin in mono and cocultured KG1a cells treated with HDACi, Api (5hr, 1μM).

Supplement Fig. S4A, CRISPR-editing of ACSS2 at S30 site (exon 1) with western blot of HS-5 single cell clones expanded of transfected cells with CRISPR plasmids targeting ACSS2. Representative lysates of HS-5 ACSS2-KO clones 1-6 show no ACSS2 expression compared to HS-5 WT whereas clone 7 depicts a clone not selected for further investigation. **B,** HS-5 ACSS2-KO single cell clones (Cl1-Cl6) proliferative growth compared to HS-5 WT. **C,** Apoptosis levels of KG1a cultured with HS-5 WT or with HS-5 ACSS2-KO (clones 1 and 2). The KG1a mono and cocultures (HS-5 WT or ACSS2-KO) were treated with HDACi (Api, SAHA, or CBHA). All concentrations were at 10μM. **D,** The FCCP-stimulated OCR was used to calculate spare respiratory capacity, the difference between maximal respiration (the maximal energy demand the leukemic cells can achieve) and basal respiration (the energy demand under basal conditions). Most of the basal respiration is coupled to ATP-linked respiration**.** These data complement the representative data

shown in Fig. 4A. **E,** Leukemic proliferative growth with increasing acetate (0-50 μM, 48h) dose is induced in KG1a, OCI-AML3, and K562.

Supplement Fig. S5A, A representative flow cytometry histogram of KG1a cells treated with acetate (10μM) or with Api (HDACi, 1μM) 48h is shown. The intracellular expression of H3 and H3K9ac was assessed by flow cytometry. **B,** Positive cells were determined by a shift in fluorescence intensity relative to unstained cells. The percentage of positive cells were averaged for four replicates for H3 and H3K9ac measurements. Paired T-test was used to calculate the significance difference between untreated versus acetate or HDACi treatment. Shown is the significance of increased expression of H3 (p-value <0.003) and H3K9ac (p-value <0.01) with either acetate (10µM, 48h) or HDACi (Api, 1µM, 48h) treatment relative to untreated control.

Supplement Table. S1. Clinical and mutational characteristics of AML samples used for proteomic profiling.

Supplement Table. S2. TCGA groups A and B with the clinical, cytogenetic, and molecular variables are evaluated. **A,** Characteristics of the Groups (1 and 2) defined by the levels of expression of genes in the signature are shown in the table displaying the skewness of many clinical features and characteristics of patients (see p≤0.05). Abbreviations used: white blood cell counts (WBC), French-American-British (FAB), core-binding factor (CBF), cytogenetically normal (CN), mutation (mt). **B,** The table depicts factors with a significant effect on overall survival probability for the TCGA cohort. Complex karyotype was defined by the

presence of 3 or more cytogenetic aberrations. The covariate analysis reports the stratification of Group 2 with covariates, showing the p-value of Group 2 from the Cox regression analysis. A multivariate analysis of independent factors found for the TCGA cohort. Univariate and multivariate Hazard Ratios (HR) were calculated with Cox's proportional hazard model. Abbreviations used: white blood cell count (WBC), years (yr), mutated gene (mt), and wild-type (wt).

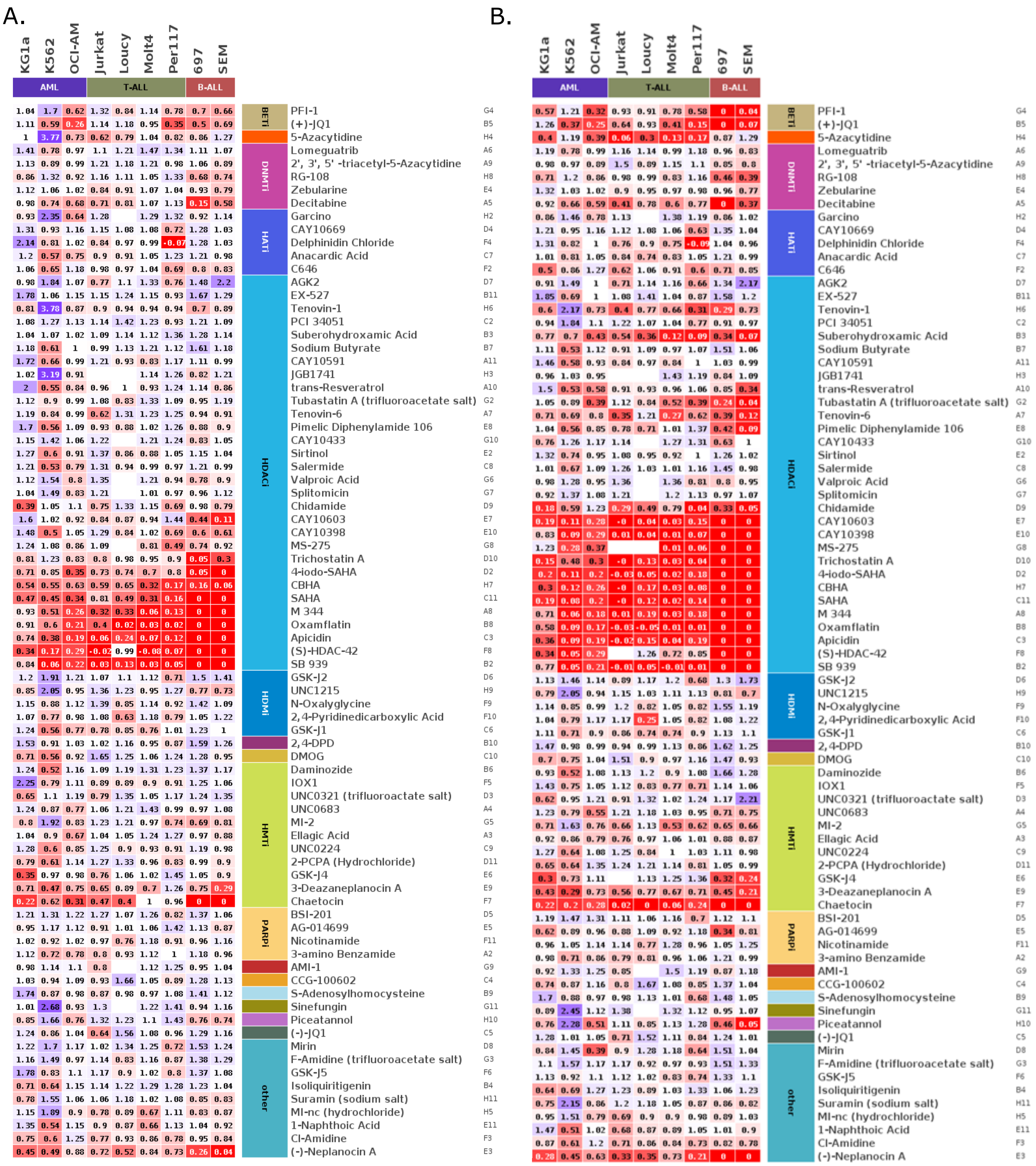

Supplement Fig. 1

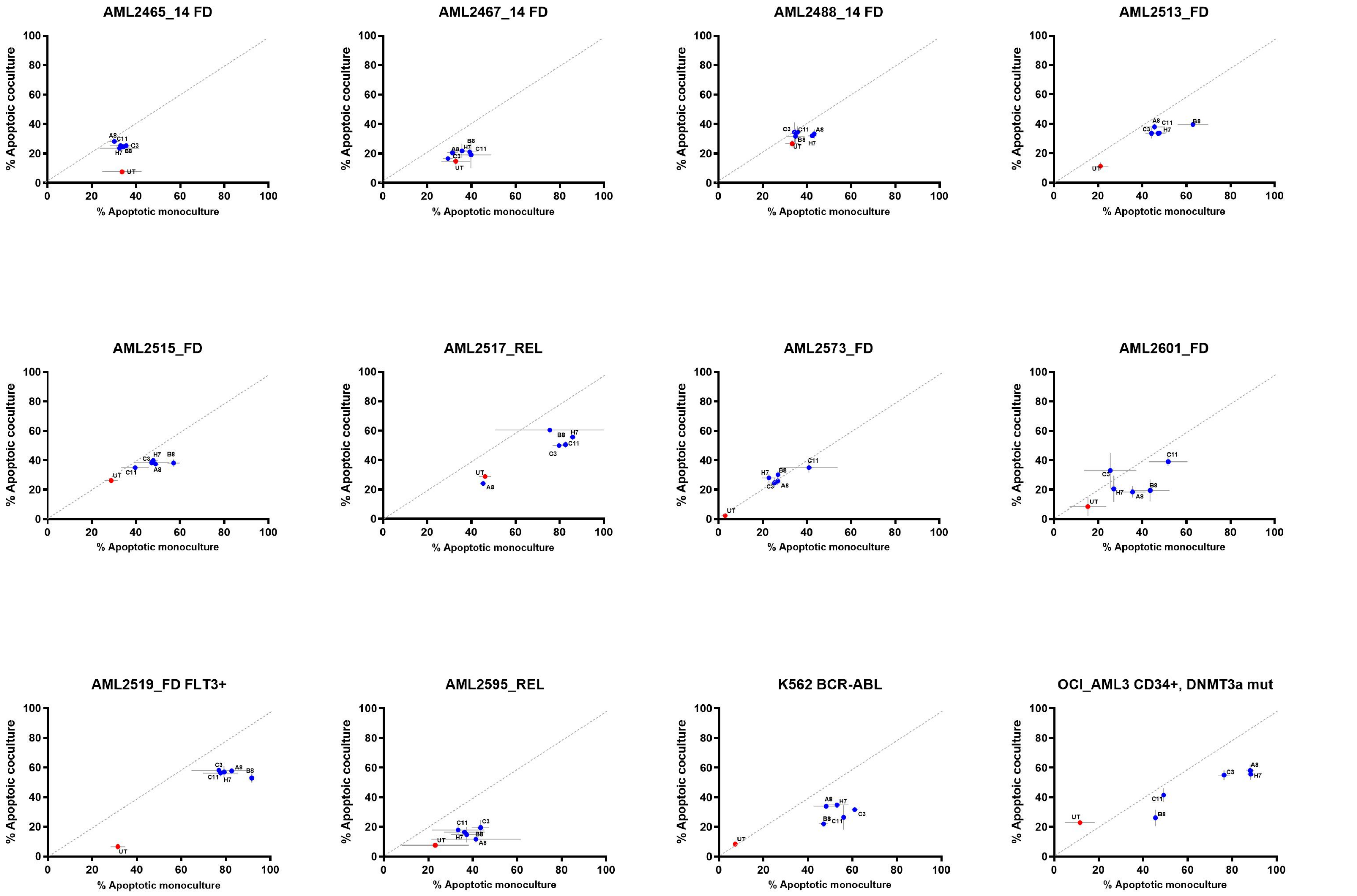

Supplement Fig. 2

A.

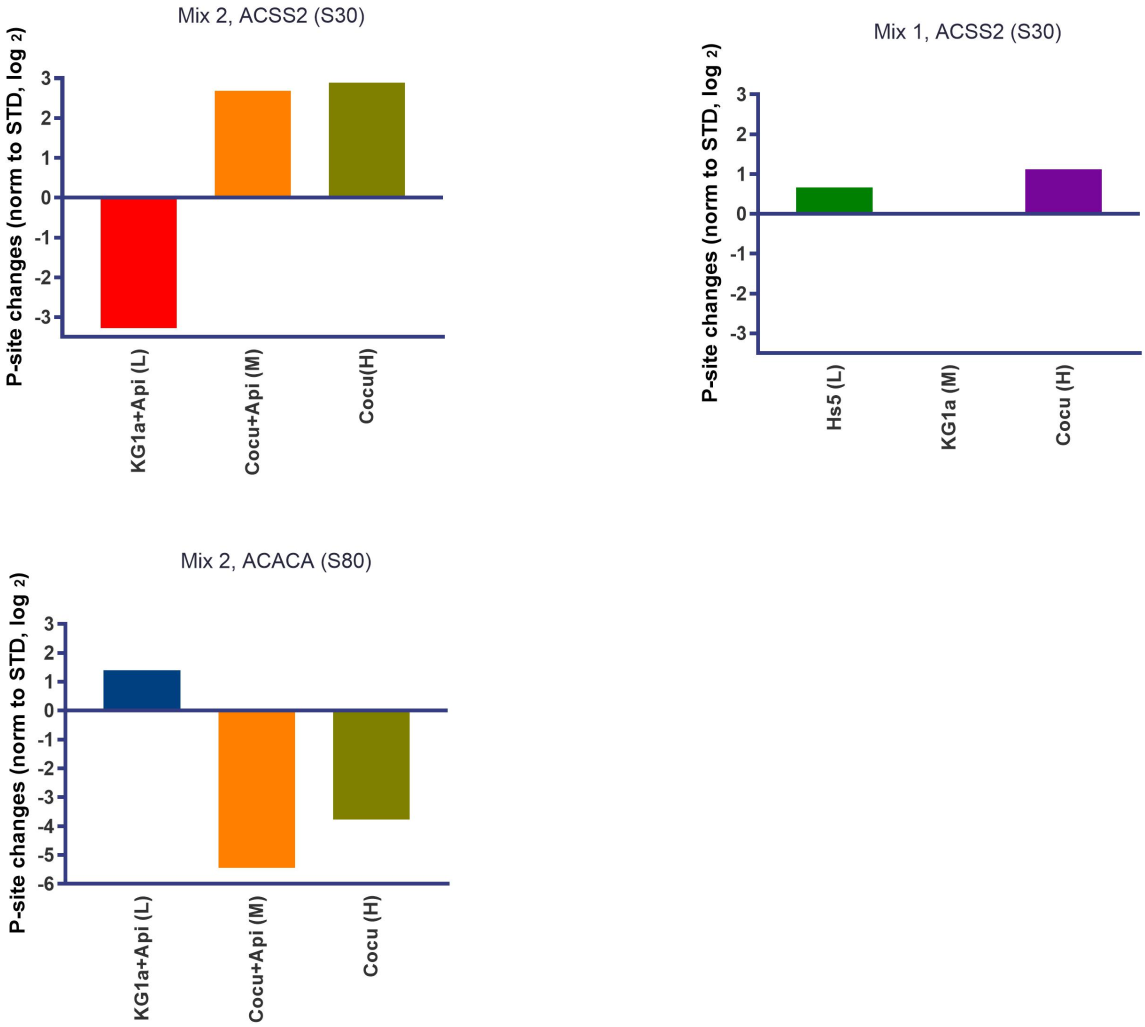

B.

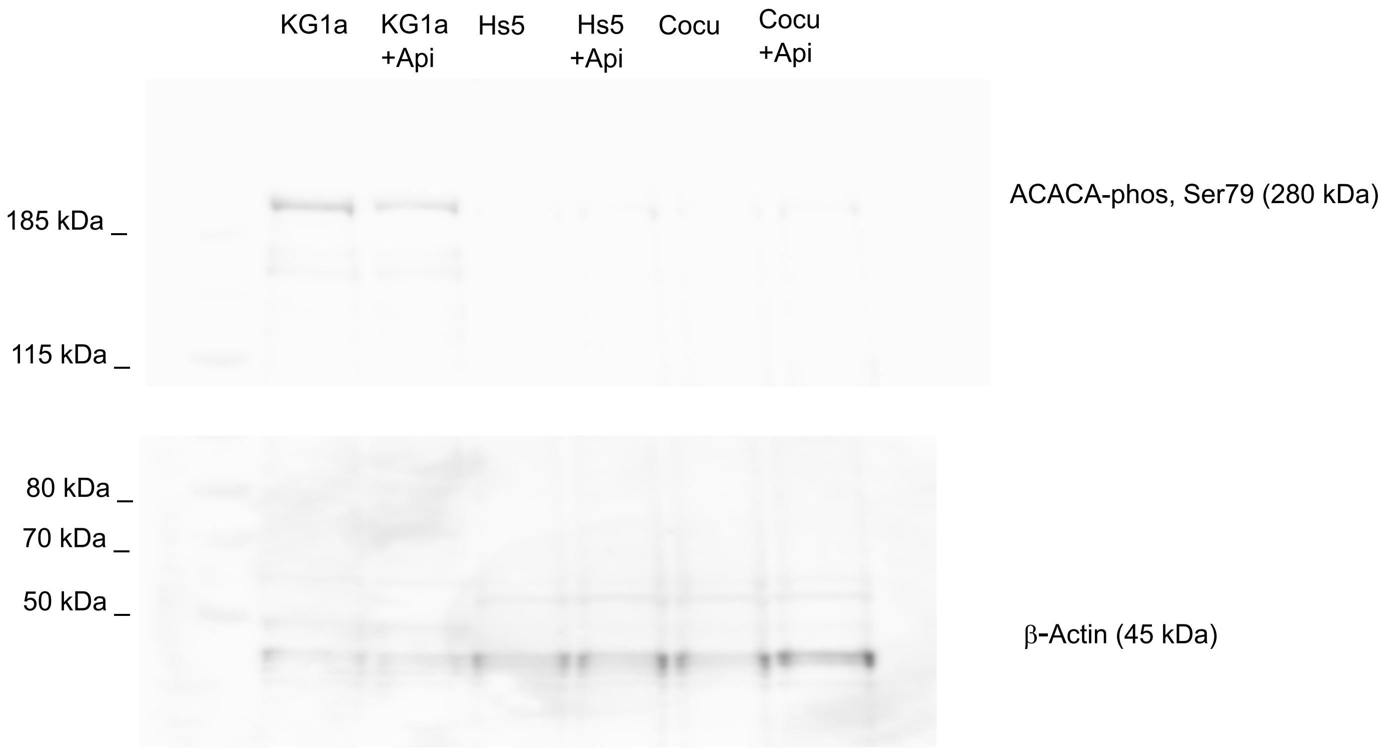

Supplement Fig. 3

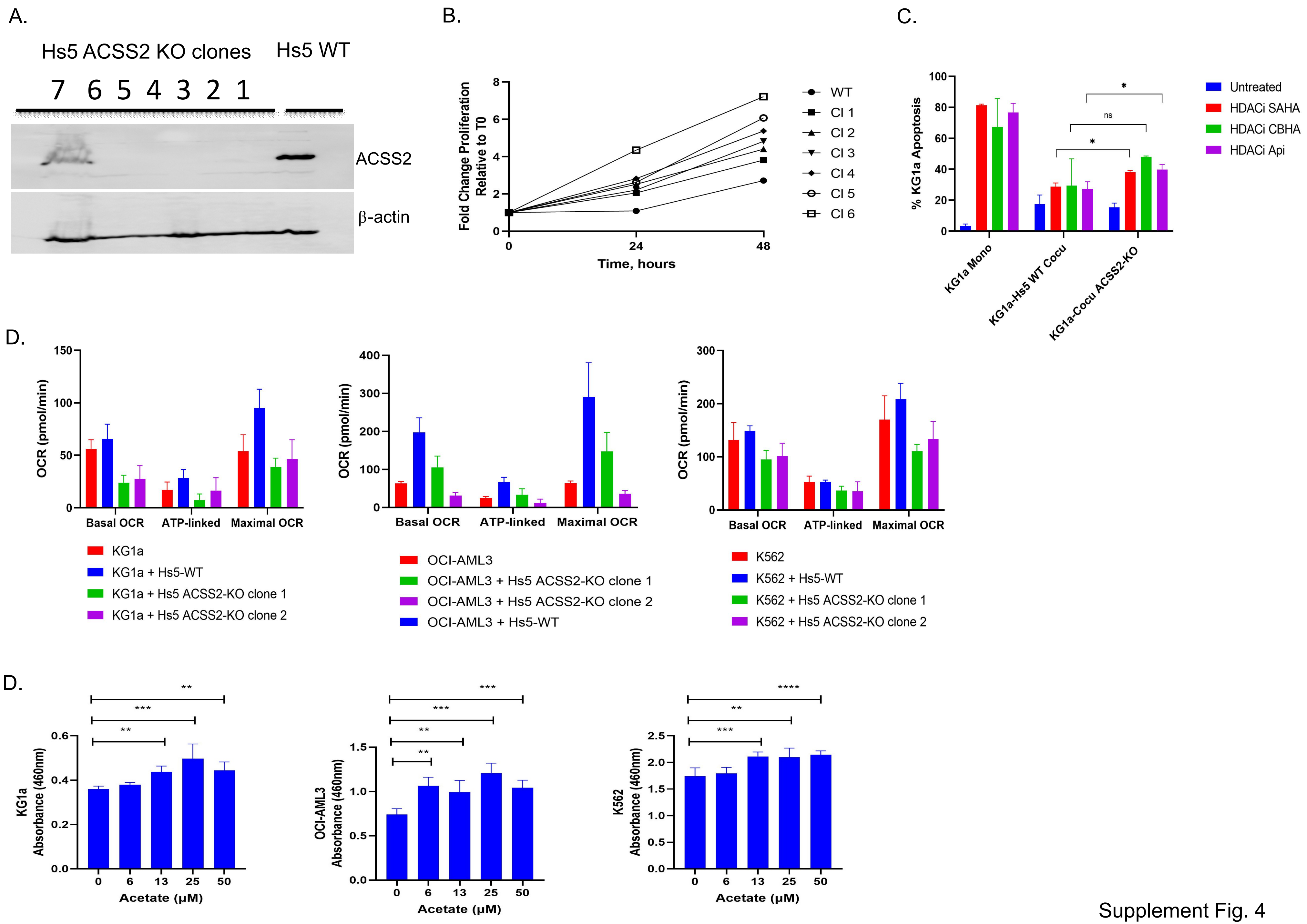

Supplement Fig. 4

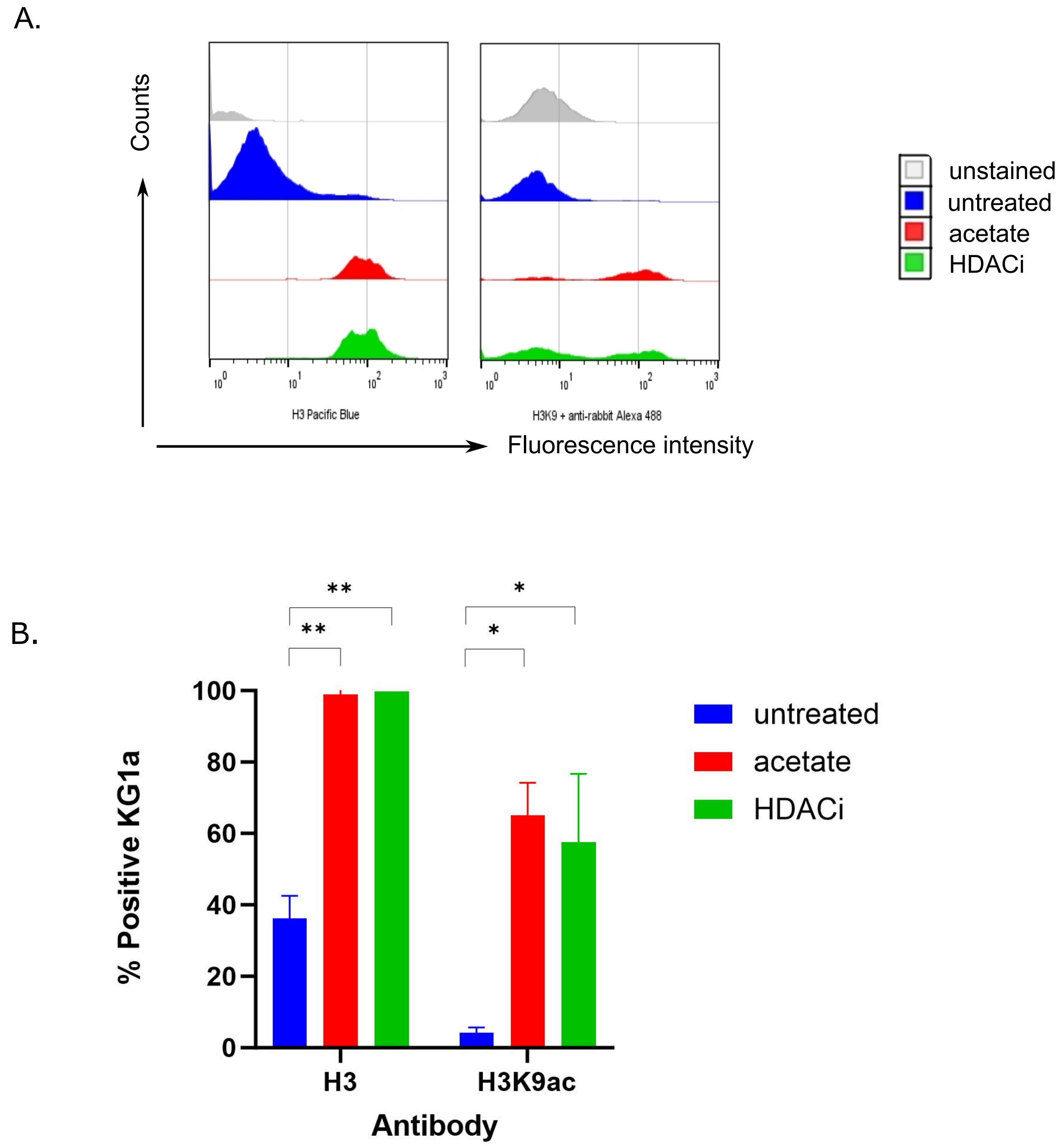

Supplement Fig. 5

A.

Clinical background summary of AML-derived bone marrow aspirate samples used for proteomic profiling (AML14).		
**AML Clinical Background**	**Type**	**Number of Patients**
Gender		
	female	6
	male	8
Age, years		
	mean	71
	range	45-87
FAB classification		
	M1	2
	M4	2
	M5a	4
	Not available	6

B.

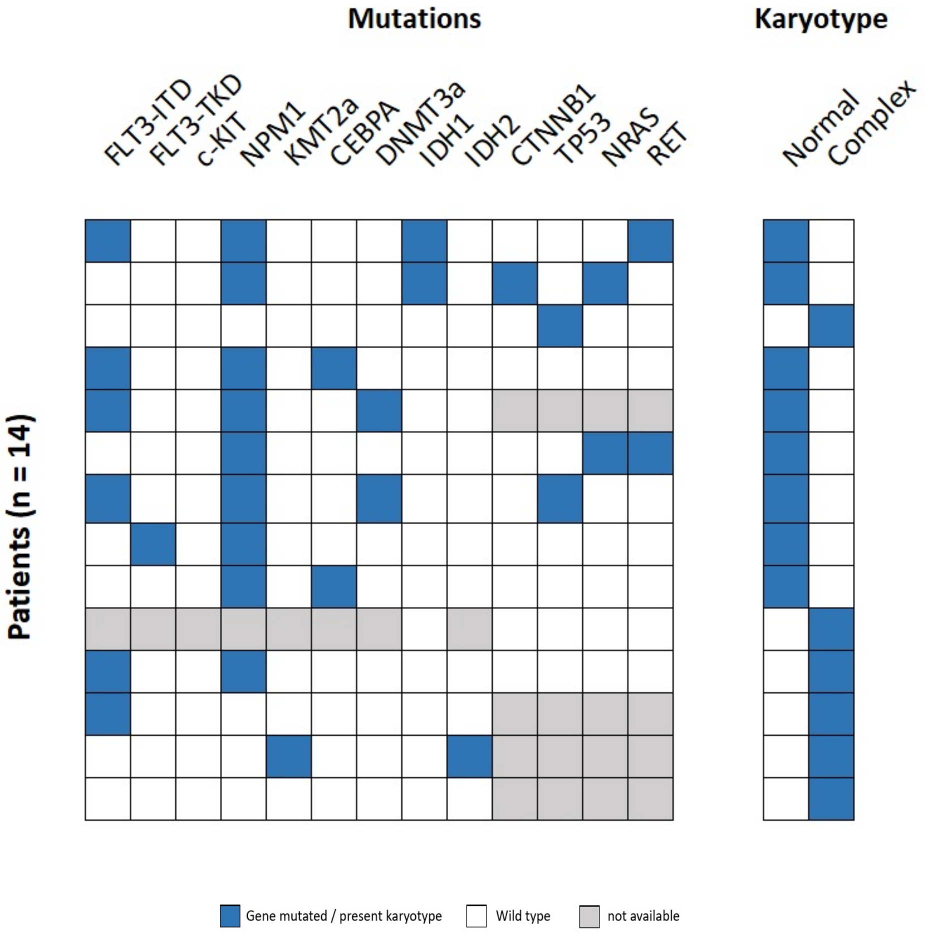

A.

Clinical Characteristics	Group 1 (N=96) ACSS1/2-low	Group 2 (N=60) *ACSS1/2*-high	p-value
**Sex**			0.223 [1]
F	48 (50.0%)	24 (40.0%)	
M	48 (50.0%)	36 (60.0%)	
**FAB**			0.100 [1]
M0	8 (8.3%)	8 (13.3%)	
M1	26 (27.1%)	18 (30.0%)	
M2	28 (29.2%)	10 (16.7%)	
M4	25 (26.0%)	9 (15.0%)	
M5	7 (7.3%)	11 (18.3%)	
M6	0 (0.0%)	1 (1.7%)	
M7	1 (1.0%)	2 (3.3%)	
nc	1 (1.0%)	1 (1.7%)	
**Age (years)**			**< 0.001** [2]
Count	96	60	
Median	55	66.5	
Q1,Q3	40.7, 62.2	57.7, 75.0	
**WBC (/nL)**			**0.01** [2]
Count	96	60	
Median	29.5	12.2	
Q1,Q3	8.3, 68.6	2.9, 41.0	
**Cytogenetic Classes**			**0.001** [1]
BCR::ABL1	1 (1.0%)	2 (3.3%)	
CBFB::MYH11	9 (9.4%)	1 (1.7%)	
Complex Cytogenetics	5 (5.2%)	16 (26.7%)	
Intermediate Risk Cytogenetic Abnormality	11 (11.5%)	9 (15.0%)	
MLL translocation, poor risk	3 (3.1%)	0 (0.0%)	
MLL translocation, t(9;11)	1 (1.0%)	0 (0.0%)	
N.D.	2 (2.1%)	1 (1.7%)	
Normal Karyotype	55 (57.3%)	24 (40.0%)	
Poor Risk Cytogenetic Abnormality	3 (3.1%)	6 (10.0%)	
RUNX1::RUNX1T1	6 (6.2%)	1 (1.7%)	
**Molecular risk classification**			**< 0.001** [1]
Good	15 (15.6%)	2 (3.3%)	
Intermediate	63 (65.6%)	29 (48.3%)	
N.D.	2 (2.1%)	1 (1.7%)	
Poor	16 (16.7%)	28 (46.7%)	
**Complete Remission**			**0.01** [1]
no			
yes	41 (42.7%)	13 (21.7%)	
ACSS1 expression			**0.001** [2]
Count	96	60	
Median	-0.07	0.17	
Q1,Q3	-0.40, 0.26	-0.20, 0.54	
ACSS2 expression			**0.01** [2]
Count	96	60	
Median	-0.15	0.205	
Q1,Q3	-0.65, 0.25	-0.37, 0.53	
ACACA expression			**0.0012** [2]
Count	96	60	
Median	0.12	-0.155	
Q1,Q3	-0.29, 0.48	-0.57, 0.28	
**Mutations**			
TP53			**< 0.001** [1]
mt	2 (2.1%)	11 (18.3%)	
wt	94 (97.9%)	49 (81.7%)	
NPM1			**0.008** [1]
mt	37 (38.5%)	11 (18.3%)	
wt	59 (61.5%)	49 (81.7%)	
WT1			**0.010** [1]
mt	10 (10.4%)	0 (0.0%)	
wt	86 (89.6%)	60 (100.0%)	
FLT3			**0.011** [1]
mt	34 (35.4%)	10 (16.7%)	
wt	62 (64.6%)	50 (83.3%)	
STAG2			**0.05** [1]
mt	6 (6.2%)	0 (0.0%)	
wt	90 (93.8%)	60 (100.0%)	

1. Pearson's Chi-squared test
2. Kruskal-Wallis rank sum test

B.

Dependent Survival	Number (%)	HR (univariable)	HR (multivariable)
Group 1 (*ACSS1/2*-low)	96 (61.5)	-	-
Group 2 (*ACSS1/2*-high)	60 (38.5)	2.07 (1.44-2.99, **p<0.001**)	1.73 (1.13-2.63, **p=0.011**)
age [18-59]	79 (50.6)	-	-
age (60-88]	77 (49.4)	2.71 (1.87-3.95, **p<0.001**)	2.35 (1.54-3.60, **p<0.001**)
WBC low (≤80/nL)	130 (83.3)	-	-
WBC high (>80/nL)	26 (16.7)	1.48 (0.93-2.36, p=0.097)	2.01 (1.21-3.35, **p=0.007**)
Normal	132 (86.3)	-	-
Complex	21 (13.7)	1.88 (1.15-3.08, **p=0.012**)	1.37 (0.70-2.70, p=0.359)
TP53 wt	143 (91.7)	-	-
TP53 mt	13 (8.3)	4.00 (2.19-7.28, **p<0.001**)	2.46 (1.09-5.55, **p=0.030**)
FLT3 wt	112 (71.8)	-	-
FLT3 mt	44 (28.2)	1.43 (0.97-2.13, p=0.074)	1.70 (1.08-2.67, **p=0.022**)
DNMT3A wt	113 (72.4)	-	-
DNMT3A mt	43 (27.6)	1.44 (0.97-2.14, p=0.071)	1.39 (0.91-2.11, p=0.130)

Supplment Table 2
